# Paneth Cell-Rich Regions Separated by a Cluster of Lgr5+ Cells Initiate Crypt Fission in the Intestinal Stem Cell Niche

**DOI:** 10.1371/journal.pbio.1002491

**Published:** 2016-06-27

**Authors:** Alistair J. Langlands, Axel A. Almet, Paul L. Appleton, Ian P. Newton, James M. Osborne, Inke S. Näthke

**Affiliations:** 1 Cell and Developmental Biology, School of Life Sciences, University of Dundee, Dundee, United Kingdom; 2 School of Mathematics and Statistics, University of Melbourne, Victoria, Australia; Friedrich Miescher Institute, SWITZERLAND

## Abstract

The crypts of the intestinal epithelium house the stem cells that ensure the continual renewal of the epithelial cells that line the intestinal tract. Crypt number increases by a process called crypt fission, the division of a single crypt into two daughter crypts. Fission drives normal tissue growth and maintenance. Correspondingly, it becomes less frequent in adulthood. Importantly, fission is reactivated to drive adenoma growth. The mechanisms governing fission are poorly understood. However, only by knowing how normal fission operates can cancer-associated changes be elucidated. We studied normal fission in tissue in three dimensions using high-resolution imaging and used intestinal organoids to identify underlying mechanisms. We discovered that both the number and relative position of Paneth cells and Lgr5+ cells are important for fission. Furthermore, the higher stiffness and increased adhesion of Paneth cells are involved in determining the site of fission. Formation of a cluster of Lgr5+ cells between at least two Paneth-cell-rich domains establishes the site for the upward invagination that initiates fission.

## Abstract 2

Crypt fission—a process responsible for normal intestinal growth and for the formation of adenomas —is governed by differential adhesion, stiffness, and proliferation of Lgr5+ cells and Paneth cells in the intestinal stem cell niche.

## Author Summary

The intestinal tract undergoes many changes during development, and after birth it has to significantly elongate and widen in order to increase the surface area for absorption. Crypt fission is a key process in intestinal tissue expansion and is also involved in adenoma growth. Despite the importance of crypt fission, the mechanisms controlling it are poorly understood. Understanding how crypt fission is regulated in normal tissue can help us to determine how the process changes in cancer. Here, we describe cellular behaviour during crypt fission. We identify a specific cellular arrangement in the intestinal stem cell niche that is associated with crypt fission and reveals insights into the mechanisms controlling crypt fission. There are two different cell types at the crypt base, Lgr5+ and Paneth cells, which play distinct roles in this process. We find that both their location and differences between them in proliferation, stiffness, and adhesion are important for fission. Based on our data, we propose a model in which stiffer and more adhesive Paneth cells are necessary to shape the crypt base and establish where fission occurs, whereas softer Lgr5+ cells allow shape changes and proliferation to expand newly formed crypts. Our model is an important step in understanding how crypt fission is initiated in normal tissue and provides a framework to understand how the process changes in tumorigenesis.

## Introduction

**Fig 1:**
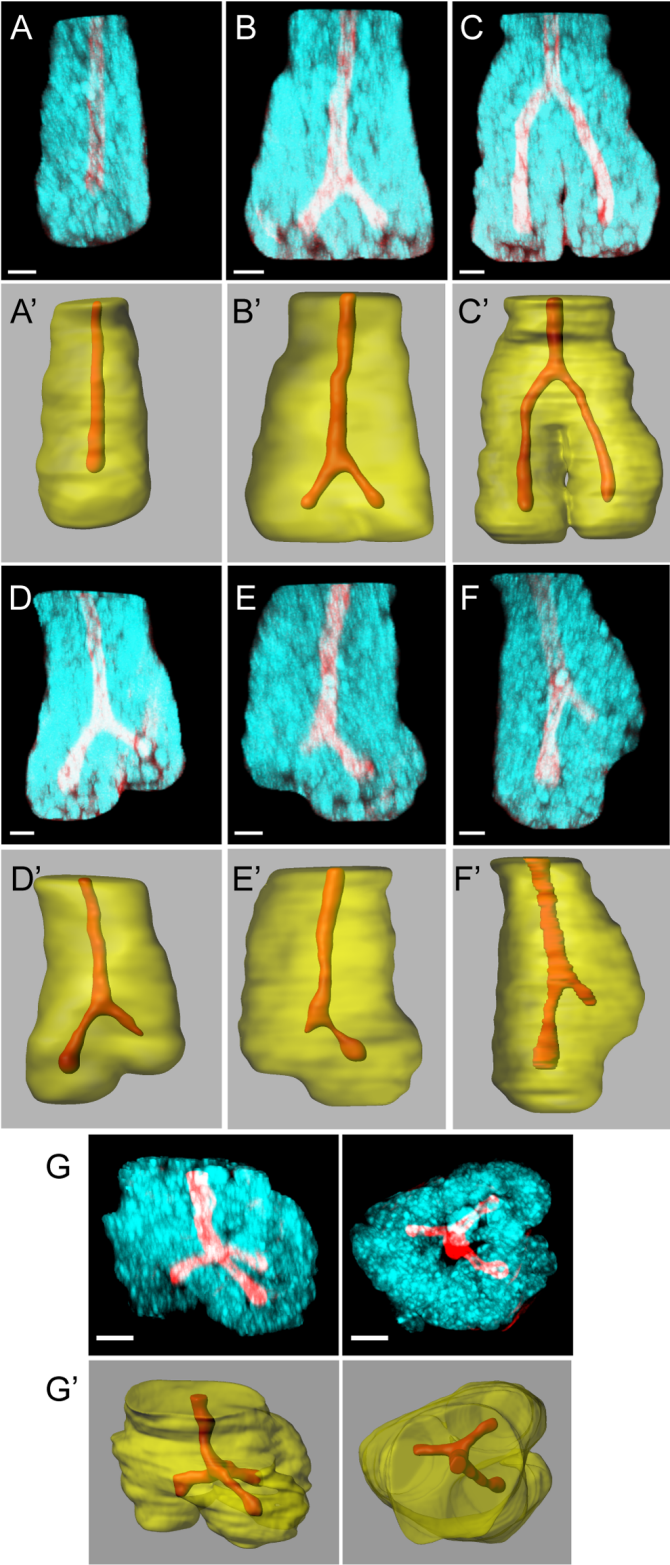
Types of fission observed in mouse small intestine. Immunofluorescence images of small intestinal crypts stained with Hoechst (cyan) and Phalloidin (red; A–G) reveal crypt structure in three dimensions. Imaris-rendered surfaces of the same images show the crypt lumen (red) and crypt wall (transparent yellow; A′–G′). (A) A single crypt not undergoing fission has a single straight lumen. (B) An early-stage symmetrical fission is characterised by a bifurcation of the lumen near the crypt base. (C) In a late-stage symmetrical fission, the bifurcation of the lumen is near the top of the crypt and the outer crypt wall has separated to form two daughter crypts. (D–F) Asymmetrical fission has two branches of unequal length and can initiate near the crypt base (D, E), or above the crypt base (F) in a budding process. (G) Triple fission is the trifurcation of the lumen into three daughter crypts; shown here from the side (left panel) and the top (right panel). Bar = 10 μm.

The structures of many adult epithelia arise from branching events during development. For instance, the organisation of adult lung, kidney, and mammary epithelia arises by branching of epithelial tubes that ceases once the tissue is fully formed. A related but distinct form of branching is important in the gut, where the crypts of Lieberkühn divide in a fissioning process to elongate and widen the intestinal tract during postnatal development [1]. Crypt fission involves the division of a single crypt into two daughters (Fig 1). The incidence of crypt fission is highest in young animals and decreases with age but does not completely stop [2]. Importantly, crypt fission is reactivated in cancer and drives the clonal expansion of mutant crypts in adenoma [3–7]. For instance, polyps in *Apc*^*Min/+*^ mice and in familial adenomatous polyposis (FAP) patients are initiated by and expand through crypt fission [8–10]. Many reports describe the importance of crypt fission in growth of healthy and cancerous tissue; however, a detailed understanding of the underlying mechanisms is lacking.

The crypt base in the small intestine contains two major cell types: Lgr5+ cells, including stem cells; and secretory Paneth cells. Producing two crypts of normal size from one crypt requires an increase in the number of Paneth and stem cells between fission events. However, there is currently no consensus about the requirement of either of these cell types for the formation of new crypts. It has been proposed that crypt fission is driven by an expansion of the stem cell pool [11]. On the other hand, budding of new branches from intestinal organoids, a process related to fission, has been proposed to require Paneth cells [12–14]. However, the ability of intestinal tissue lacking Paneth cells to repair after injury questions the requirement of Paneth cells in this process [14,15].

To complicate matters further, recent reports have challenged the classical model of crypt fission as a bifurcation of a parental crypt, and instead propose that it occurs as “asymmetric budding,” with daughter crypts formed by budding from a larger parental crypt [16]. In intestinal organoids, new crypts can also form by budding from a spherical structure [12–14,16].

To understand the processes that govern normal fission, we utilised 3D imaging of whole mount tissue [17]. We examined crypts undergoing fission at high resolution and detected multiple types of fission during normal postnatal development. Monitoring Lgr5+ and Paneth cells, we found a cluster of Lgr5+ cells at the earliest stages of fission. This cluster marks the site of the bifurcation that initiates fission. Using whole tissue and organoids, we determined that Paneth cells have more β4 Integrin on their basal surface and are more adherent to laminin, a major component of the basement membrane. We also find that adhesion by β4 Integrin is important for normal fission. Computational models of tissue dynamics support roles of both Paneth and Lgr5+ cells in fission. We propose that Paneth cells promote deformation of the crypt base. A cluster of Lgr5+ cells forms between two Paneth-cell-rich areas, creating an area with lowered adhesion and mechanical stiffness that permits tissue buckling.

## Results

### Imaging Whole Mount Tissue at High Resolution in 3D Reveals Distinct Stages of Crypt Fission

Crypt fission has been studied mostly using sectioned tissue or isolated crypts. Details of the earliest stages of fission are difficult to identify by either of these approaches. Sectioned tissue provides limited information about the 3D relationship of tissue structures. In isolated crypts stained only with DAPI [16], information about the position of daughter crypt bases relative to surrounding connective tissue is lost. Here, we use whole mount mouse tissue to identify mechanisms involved in crypt fission. High-resolution multiphoton confocal microscopy allowed visualisation of the 3D structure of crypts undergoing fission in tissue stained to visualise F-actin and nuclei (Fig 1). Previously unknown details about cell and tissue arrangements at all stages of fission were revealed.

Intestinal crypts have a straight lumen extending from the stem cell compartment at the base toward the opening facing the gut lumen (Fig 1A). Bifurcation of the crypt lumen marks fissioning crypts. To identify specific features of fission, we first documented different stages of fission based on the position of the bifurcation relative to the crypt base. The earliest stages are characterised by widening of the crypt base and the appearance of a branch point between two very short prospective daughter crypts (Fig 1B). As fission propagates, the bifurcation ascends upward from the crypt base (Fig 1C). Fission is complete when the bifurcation reaches the top of the crypt producing two new, separated daughter crypts.

The lumens of daughter crypts can be of equal or unequal length, distinguishing symmetric (Fig 1B and 1C) and asymmetric fissions (Fig 1D–1F), respectively. Asymmetric fission occurs when the initial bifurcation is not symmetrically placed (Fig 1D and 1E) or when fission is initiated above the crypt base (Fig 1F). Previously, fissions producing unequal daughter crypts have been referred to as “asymmetric budding” [16]. However, this classification relied on imaging isolated crypts released from the underlying connective tissue prior to fixation and imaging. Our in situ approach allows us to distinguish between asymmetric fission that results from a branch forming on the side of a crypt (i.e., away from the crypt base, which we define as budding) and asymmetric placement of the bifurcation at the crypt base, which we define as asymmetric fission. Using our method, we also observed examples of trifurcating crypts (Fig 1G) producing three daughter crypts. We found that such “triple fission” can be symmetric (Fig 1G), with three daughter crypts of equal length, or asymmetric (S1 Fig), with daughter crypts of different lengths.

### Incidence of Fission Varies with Age and Along the Proximal–Distal Axis of the Intestinal Tract

Observation of different types of fission raises the question of their prevalence in normal development. We therefore counted and scored fission in mouse intestinal tissue. Three regions in each of the small intestine and the colon were examined from mice aged 2, 3, 4, 5, 6, and 10 wk. Asymmetric fission was defined visually as fission with daughter crypts differing by approximately 25% in length; both budding and asymmetric fissions were counted as asymmetric. One hundred to 200 crypts were scored in each region from three individual animals in each age group.

**Fig 2:**
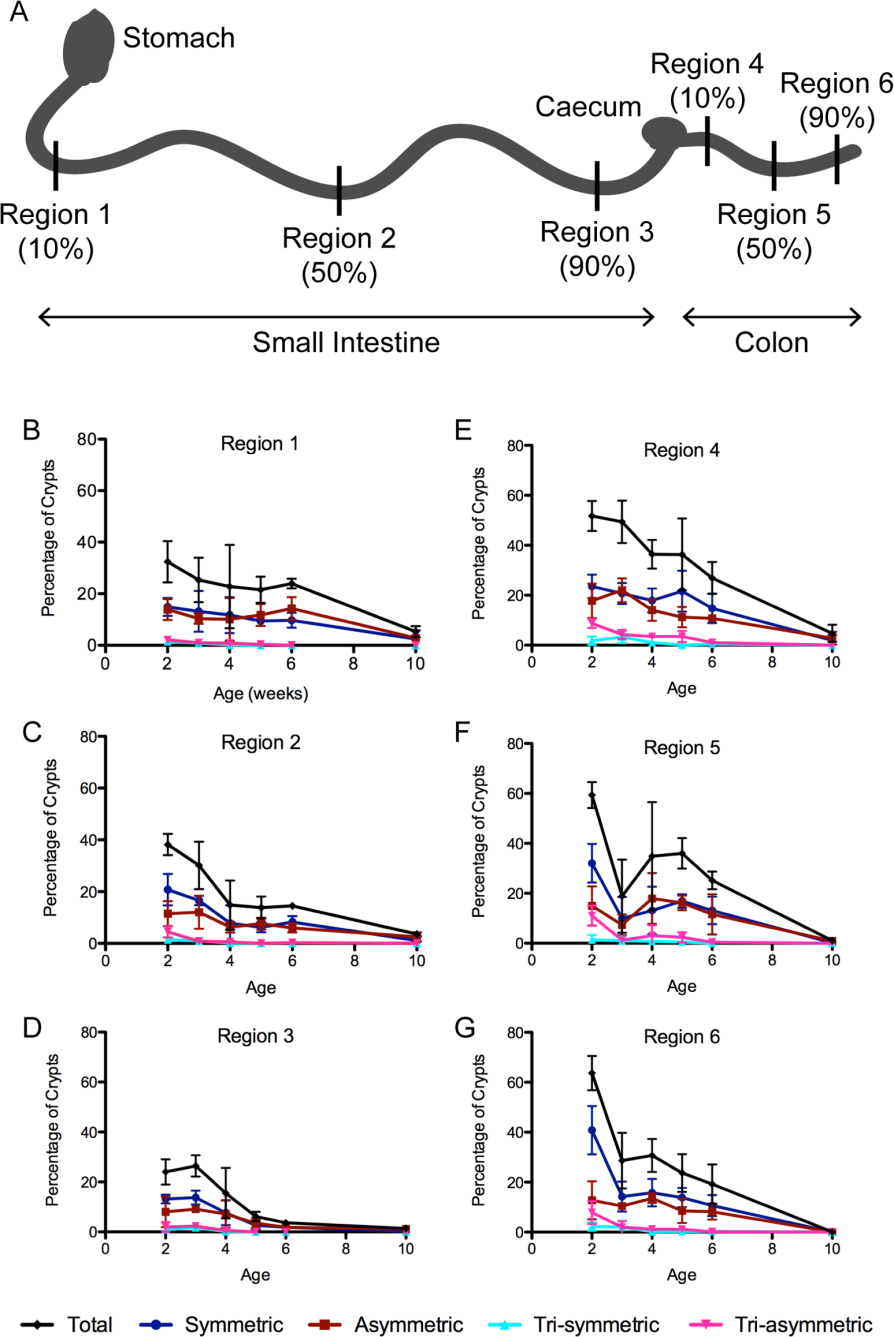
Incidence of crypt fission during postnatal development. Crypts undergoing fission were counted in all regions (A) of the small intestine (B–D) and colon (E–G) isolated from mice aged 2, 3, 4, 5, 6, and 10 wk. Graphs show percentage of crypts undergoing fission and the proportions of different types of fission as defined in Fig 1. (A) Schematic diagram of the regions of the small intestine and colon, as defined in [8]: Regions 1 (B) and 4 (E) are at 10% of the proximal-distal distance along the small intestine and colon, respectively; Regions 2 (C) and 5 (F) at 50%; and Regions 3 (D) and 6 (G) at 90%. Tissue from three mice was analysed for each age group, with 100–200 crypts counted in each region in each mouse. Error bars show standard deviations between mice. Values for percentage crypt fission are shown in S1 Table, and underlying data for panels B–G can be found in S1 Data.

Consistent with previous reports [1,2,11,18–20], fission was most prevalent in rapidly growing young mice (Fig 2, S1 Table and S1 Data). The highest incidence of fission was at 2 wk of age, when approximately 40% of all crypts in region 2 (jejunum) and 60% of all crypts in region 5 (middle colon) were fissioning. These numbers are similar to those reported in isolated crypts [2], but are higher than reported in tissue sections [11]. Sectioned tissue permits identification of fission events only when they are orientated parallel to the sectioning plane. Since the orientation of fission is not uniform (S2 Fig), the incidence of fission is likely underestimated when using tissue sections.

Fission incidence declines with age, and by 10 wk the highest incidence was approximately 5% in regions 1 and 4. The rate of decline in fission varies between different regions but is most pronounced in the distal regions of both the small intestine and colon, where only approximately 1% of crypts undergo fission in 10-week-old animals (Fig 2, S1 Table, and S1 Data). Symmetric and asymmetric fissions occur with similar frequencies throughout development and in all regions. Triple fissions are rare, except in rapidly growing tissue of 2-week-old animals when 12.4% of all crypts in region 5 are undergoing triple fission. No triple fissions were detected in 10-week-old mice. Triple fission may only be a feature of rapidly growing tissue in young mice, explaining why it has not been identified in normal tissue previously.

### Changes in Cell Patterning Mark the Onset of Fission

**Fig 3:**
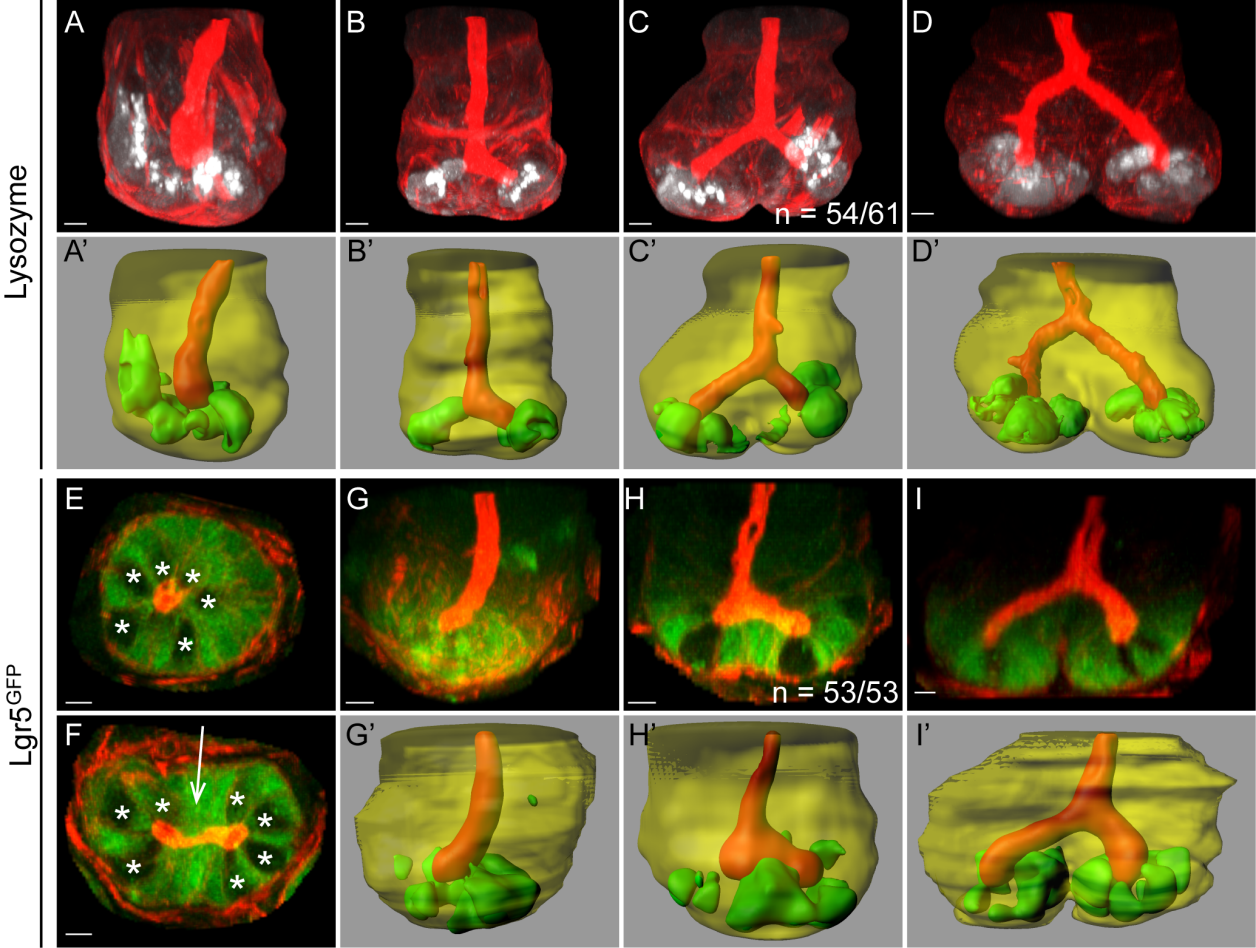
Cell sorting during fission. The localisation of Paneth cells and Lgr5+ stem cells changes during fission, as revealed by immunofluorescence of tissue stained with Phalloidin (red; A–I) and Imaris-rendered surfaces showing the crypt lumen (red) and crypt wall (transparent yellow; A′–D′, G′–I′). Antibodies against lysozyme (A–D, white; A′–D′, green) and GFP (E–I, green) were used to visualise Paneth cells and *Lgr5*^*GFP*^+ cells, respectively. Imaris-rendered surfaces for Paneth and Lgr5+ cells were generated by thresholding. (A) Paneth cells are located at the base of single crypts in the stem cell niche. Paneth cells in early fissioning crypts (B, C) are excluded from the central region that marks where bifurcation initiates. (D) In a late fissioning crypt (i.e., the bifurcation point is above the stem cell compartment), Paneth cells are restricted to the base of the two daughter crypts. (E) Optical section of a single *Lgr5*^*GFP*^+ crypt reveals the alternating pattern of Lgr5+ and Paneth cells (visible as GFP- cells, indicated by *). (F) Optical section of an *Lgr5*^*GFP*^+ crypt in early fission reveals Lgr5+ cells in a cluster between the lumens of the two prospective daughter crypts (arrow). (G) *Lgr5*^*GFP*^+ cells are located at the crypt base in a single crypt. (H) An early fissioning crypt shows clustering of *Lgr5*^*GFP*^+ cells in the buckling region. (I) A side view of a late fissioning crypt shows *Lgr5*^*GFP*^+ cells are maintained at the crypt base but are lost from the bifurcation.

Visualising fission events at all stages at high resolution facilitated observing the behaviour of cells during fission. The crypt base contains at least two cell types: secretory Paneth cells and Lgr5+ cells. The arrangement of these two cell types in early fission was determined in crypts from mice expressing Lgr5^GFP^ [21] and stained with antibodies against Lysozyme to mark Paneth cells (Fig 3). In the base of single, non-fissioning crypts, Lgr5+ cells and Paneth cells are arranged in an alternating pattern (Fig 3A, 3E and 3G). At the earliest stage of fission, Paneth cells were absent from the middle of the crypt and were only found at either side of the position that marks the initiation of the bifurcation (Fig 3B and 3C). We examined 61 crypts at this stage and found that Paneth cells were completely absent from the initial bifurcation site in 54 cases, and only a single Paneth cell was detectable at this site in the remaining 7 cases. The same exclusion of Paneth cells from the bifurcation was observed in fissioning crypts in human tissue (S3 Fig). Correspondingly, we found that Lgr5+ cells formed a cluster in the middle of the crypt base, and this cluster marked the region where tissue appeared to buckle upward (Fig 3F and 3H). Such a cluster was observed in all 53 examples examined. These data reveal that clustering of Lgr5+ cells creates the site where bifurcation initiates fission and that Paneth cells are excluded from this region.

At later stages of fission, Paneth cells continued to reside exclusively at the base of the daughter crypts (Fig 3D). Lgr5^GFP^ expression was lost from the cells forming the bifurcation once it had been displaced above the crypt base (Fig 3I). At this stage, both Lgr5+ and Paneth cells were restricted to the base of the two new daughter crypts. Based on these results, we divided fission into two distinct phases. The first phase, early fission, involves a change in the distribution pattern of cells in the stem cell niche that initiates a bifurcation and establishes the base of two distinct prospective daughter crypts. The second phase, late fission, involves an expansion of the bifurcation upward to complete formation of two new crypts.

### Paneth Cells Are More Adherent than Other Crypt Cells

**Fig 4:**
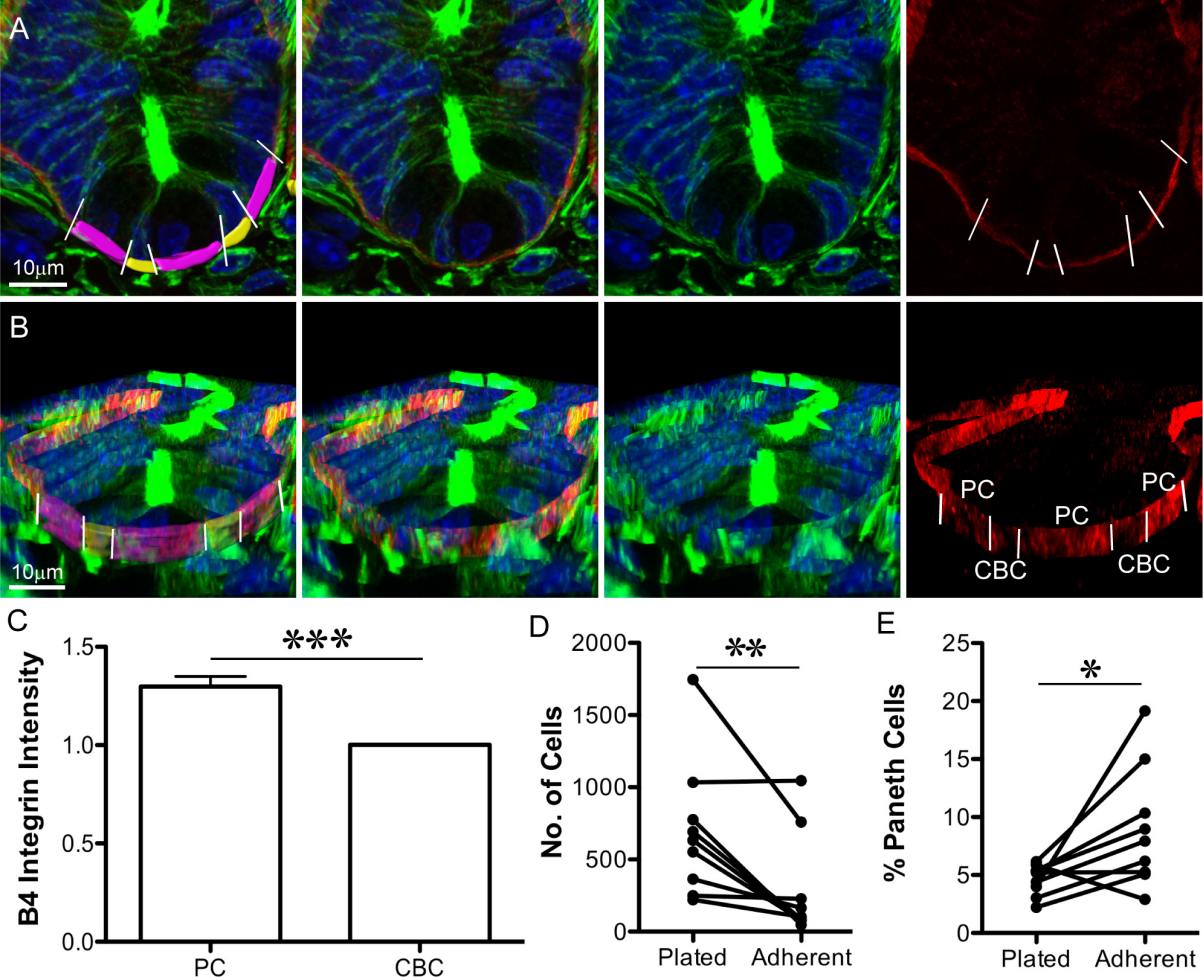
Paneth cells are more adherent to laminin than other crypt cells. (A, B) Immunofluorescence images of sectioned tissue show nuclei (Hoechst, blue), F-actin (Phalloidin, green), and β4 Integrin (red): (A) is an optical section; (B) is a 3D projection (from Imaris) of the same crypt. In the left panel, the basal surface of crypt base columnar (CBC) cells are pseudo-coloured yellow, and the basal surface of Paneth cells are pseudo-coloured purple. Paneth cells are identifiable as the large cells with round, basally placed nuclei; CBC cells are narrow and have compressed nuclei. White lines (left and right panels) indicate the boundaries between Paneth cells and their neighbours. (C) Average β4 Integrin intensity on the basal surface of Paneth cells (PC) was normalised to the average value for neighbouring cells (CBC). Left panels in (A) and (B) indicate the Imaris-rendered surfaces that were used to measure β4 Integrin signal intensity. ± SEM, *p* < 0.001 (*t* test), *n* = 59 Paneth cells (S2 Data.) (D) The number of mouse small intestinal crypt cells attached to laminin-coated surfaces before (Plated) and after (Adherent) shaking decreases (*p* = 0.0078, Wilcoxon’s paired *t* test of eight independent experiments, S3 Data). (E) The proportion of attached cells from (D) staining positive for Lysozyme increases after shaking (*p* 0.0195, Wilcoxon’s paired *t* test of eight independent experiments, S3 Data). Underlying data for panel C can be found in S2 Data, and for panels D and E in S3 Data.

The pattern of cell distribution we discovered in early fission prompted us to ask how this arrangement could promote the tissue buckling that drives fission; specifically, how differential properties of Paneth and Lgr5+ cells contribute. Buckling of the crypt wall in areas formed by the clustered Lgr5+ cells led to the hypothesis that these regions are more easily deformed than neighbouring Paneth-cell-containing areas. Paneth cells are more mechanically rigid than Lgr5+ cells [12]. However, in order to remain at the crypt base and resist the forces created by the buckling, they also have to adhere to their substrate more strongly. Attachment of epithelial cells to the underlying basement membrane is mediated by Integrins. The most common basal anchoring structures in the gut are dynamic focal adhesions formed by β1 Integrin [22], and hemi-desmosomes, which are more stable anchoring structures requiring β4 integrin [23]. We found that Paneth cells have an approximately 1.3-fold higher average signal intensity of β4 Integrin on their basal surface than crypt base columnar (CBC) cells (Fig 4A–4C, S2 Data). Together with the larger basal surface of Paneth cells, these data suggest that they have more β4 Integrin at their basal surface and therefore may attach more strongly to the basement membrane. To test this idea directly, we performed adhesion assays using isolated single cells from mouse small intestinal crypts. Specifically, we compared the adhesion of Paneth cells and other epithelial cells from intestinal crypts to laminin, the common ligand for β1 and β4 Integrin (Fig 4D and 4E and S3 Data). Cells were plated on laminin-coated substrates and allowed to adhere for 1 h. Weakly adherent cells were removed by shaking (Fig 4D). The increased proportion of Paneth cells present after shaking (Fig 4E) indicated that they were more adherent than other cells in intestinal crypts. Relatively stronger adhesion of Paneth cells may help to anchor the bases of prospective daughter crypts on either side of the Lgr5+ cell cluster and facilitate buckling. Paneth cells in intestinal polyps from *Apc*^*Min/+*^ mice do not have an increased signal intensity of β4 Integrin on their basal surfaces compared to neighbouring cells (S4 Fig, S2 Data). The aberrant fission in adenoma in this situation is consistent with a role for the differential adhesion of Paneth cells in normal fission.

If differences in adhesion to the basement membrane are a feature of normal fission, they are expected to also exist in the colon, where there are no Paneth cells. In colonic crypts, the stem cell niche contains secretory cells arranged in an alternating pattern with stem cells [24]. We found that, similar to Paneth cells in the small intestine, Muc2+ cells in the colonic stem cell niche had an increased signal intensity of β4 Integrin on their basal surfaces compared to neighbouring cells (S5 Fig). Therefore, crypt fission in the colon could also be supported by differences in adhesion of secretory cells in the stem cell niche to the basement membrane. Similarly, UEA-I+ Paneth/Goblet precursor cells have a higher β4 Integrin signal intensity (S6 Fig) on their basal surfaces than their neighbours. In mouse models lacking mature Paneth cells, such UEA-I+ secretory precursors may support fission.

### Fission in Organoids Mimics Fission in Tissue

Our tissue data suggested that differential adhesion between Paneth and Lgr5+ cells, together with a specific pattern of cell distribution, is important for fission. Testing this idea experimentally requires the ability to manipulate crypts. Intestinal organoids provide this opportunity. Organoids are epithelial structures derived from intestinal stem cells grown in matrigel that maintain many aspects of normal gut epithelial organisation, including alternating Paneth and stem cells at the crypt base [13]. Similar to the situation in tissue, crypt numbers increase with time as organoids grow.

**Fig 5:**
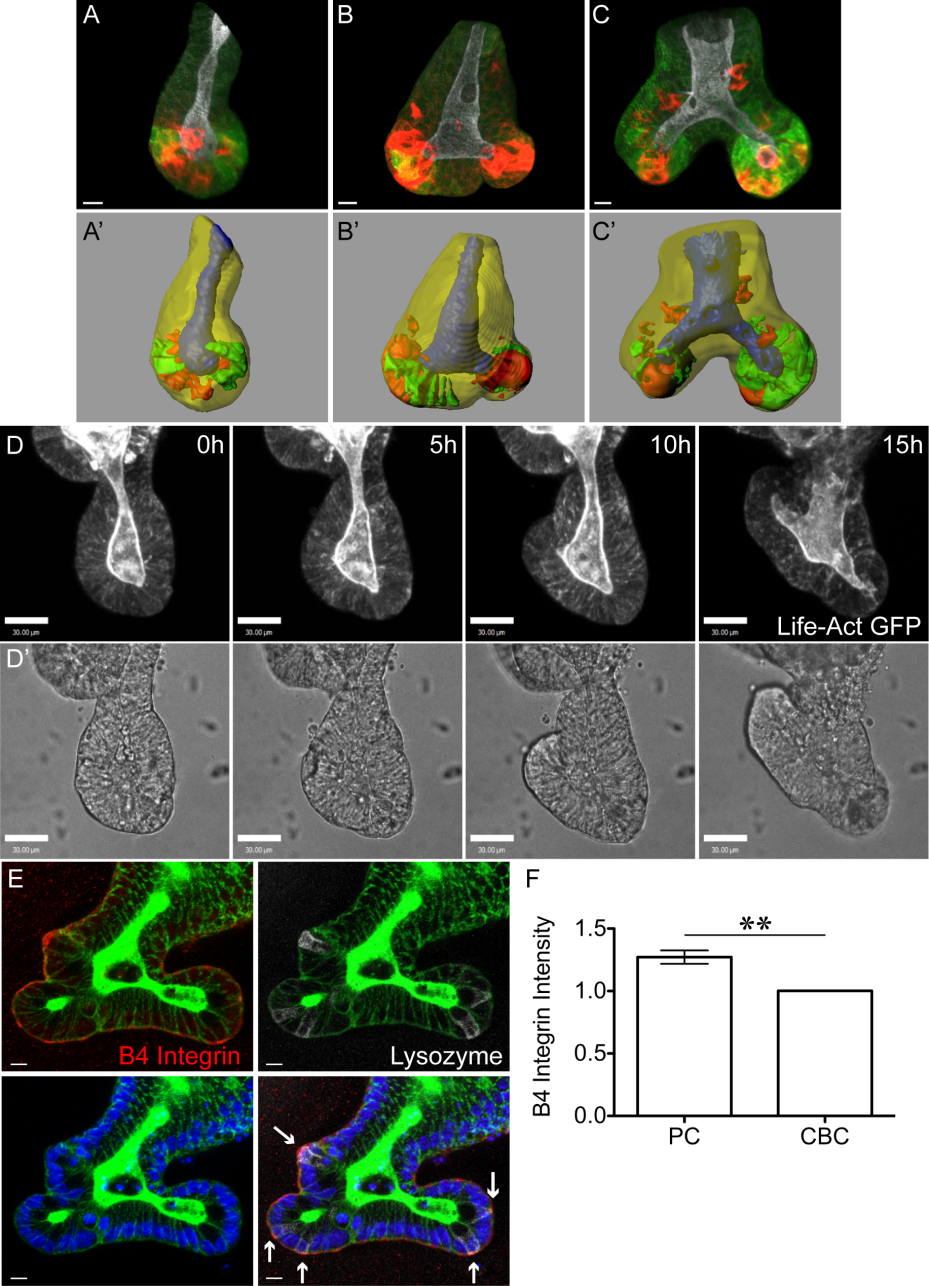
Fission in Organoids Is Similar to Fission in Tissue. Crypts in organoids were stained to visualise F-actin (Phalloidin, white), Lgr5-GFP (green), and Paneth cells (Lysozyme, red) (A–C) and corresponding Imaris-rendered surfaces of the same images prepared (crypt lumen, blue; crypt wall, transparent yellow; Lgr5-GFP+ cells, green; and Paneth cells, red A′–C′). (A) Single crypts in organoids have a straight lumen and alternating Lgr5+ and Paneth cells at the crypt base. (B, C) Fission of organoid crypts can initiate at the crypt base so that two prospective daughter crypts are formed on either side of a bifurcation. (B) Early fission is identifiable by two short prospective daughter crypts at the base of the crypt. Paneth cells are located on either side of the bifurcation, whilst Lgr5+ cells are located between the two Paneth-cell-rich regions. (C) Late fission of organoid crypts is identifiable by two longer daughter crypts sharing a common lumen above the bifurcation point. At this time, both Lgr5+ and Paneth cells are mainly restricted to the two crypt bases. (D, D′) Time lapse of Life-ActGFP expressing organoids shows a crypt base widening and two daughter crypts forming and growing in opposing directions. (E) Extended focus optical sections of an organoid stained against F-actin (Phalloidin, green), β4 Integrin (red), Lysozyme (white), and nuclei (Hoechst, blue). Arrows indicate basal membrane of Paneth cells. (F) Quantifying β4 Integrin on the basal surface of Paneth cells (PC) and their neighbouring cells (CBC) reveals β4 Integrin signal intensity is higher on Paneth cells (±SEM, *p* < 0.001, paired *t* test; *n* = 26 Paneth cells, S2 Data). Underlying data for panel F can be found in S2 Data.

The formation of new branches in organoids has been attributed to a budding-like process defined as formation of a new branch that initiates above the crypt base [16]. However, fission-like branching also occurs in organoids. To determine whether this branching process is comparable to fission in situ and to establish organoids as a suitable model to study crypt fission, we examined fixed organoids (Fig 5A–5C) and live organoids expressing LifeAct-GFP (Fig 5D). Fixed organoids contain crypts representing different stages of fission similar to those identified in tissue (Fig 5A–5C). However, in organoids, daughter crypts elongate at a 90°–180° angle (Fig 5C), rather than parallel as in tissue. The lack of physical constraint imposed by adjacent crypts and surrounding contractile cells is the most likely reason for this difference. Indeed, fissioning crypts removed from tissue before fixation exhibit a more splayed conformation (S7 Fig). The localisation of Lgr5+ and Paneth cells is similar to that identified in fissioning crypts in tissue. In early fission, Lgr5+ cells are located in the region forming the bifurcation, whilst Paneth cells are located on either side of the bifurcation (Fig 5B). In late fission, both Lgr5+ and Paneth cells are located primarily at the base of the two daughter crypts (Fig 5C). Furthermore, the average intensity of β4 Integrin on the basal surface of Paneth cells was approximately 1.3-fold higher than their neighbours (Fig 5E and 5F and S2 Data), almost identical to the difference measured in whole tissue (S8 Fig, S2 Data). Therefore, fission in organoids is similar to fission in tissue, and also may involve differential adhesion of Paneth cells and their neighbouring Lgr5+ cells. These similarities confirm organoids as a valid system to study the dynamics of fission. We therefore utilised organoids to probe mechanisms responsible for the initiation of fission. Specifically, we examined how the absolute numbers of Paneth and Lgr5+ cells per crypt, the position of Paneth cells within a crypt, and their increased adhesion to the basement membrane contribute to fission.

### Both Paneth Cells and Lgr5+ Cells Are Required for Initiation of Fission

**Fig 6:**
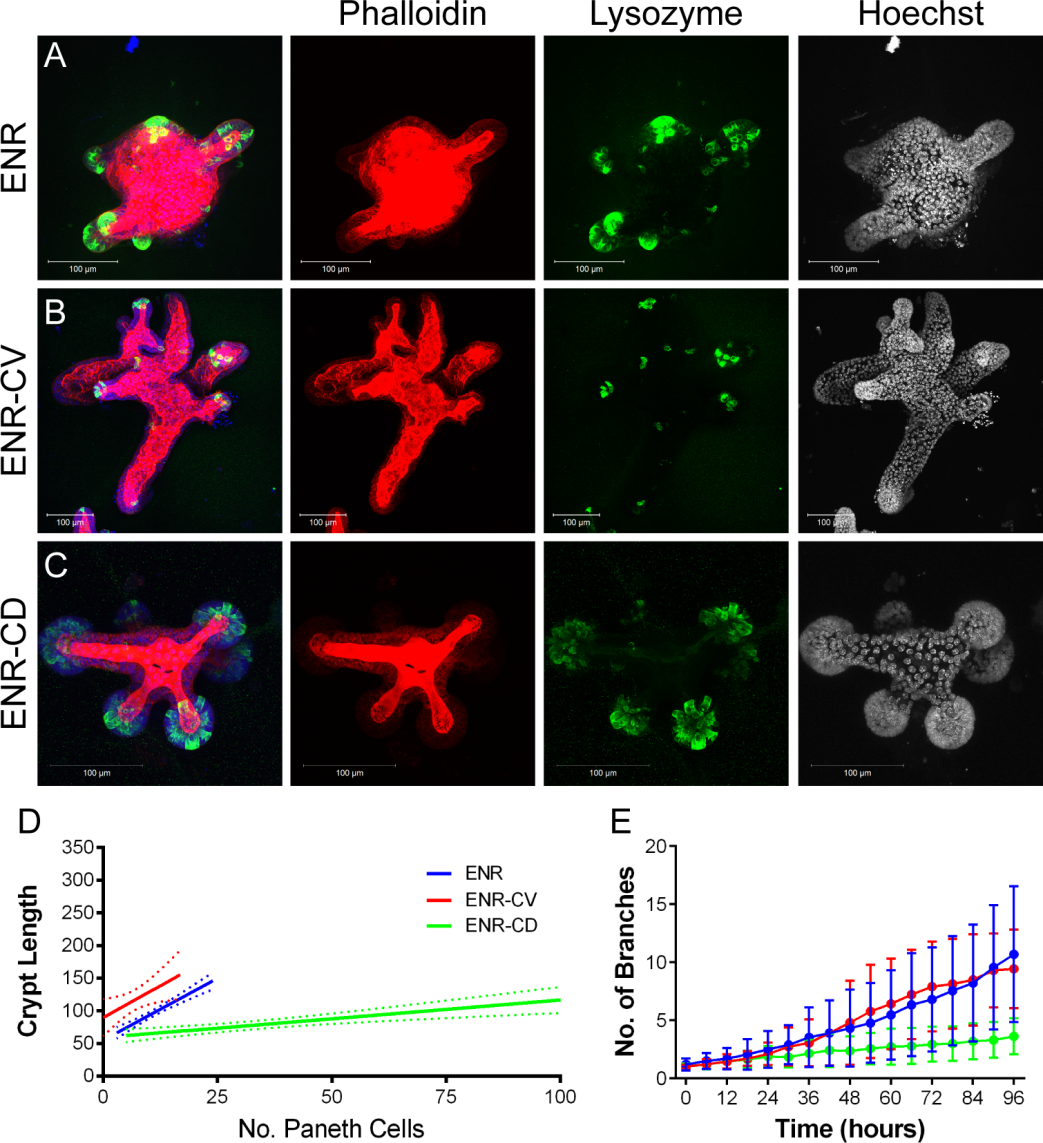
Increasing stem cell number does not increase fission. Organoids grown in control (ENR) media (A), with addition of Chiron99021 and Valproic acid to the media (ENR-CV, B), or with addition of Chiron99021 and DAPT (ENR-CD, C), were stained to visualise F-actin (Phalloidin, red), Lysozyme (green), and nuclei (Hoechst, blue, right panel). 3D projections were used to measure crypt length and Paneth cell number (D). There is a correlation between crypt length and Paneth cell number in ENR (R^2^ = 0.67) that is lost in ENR-CV (R^2^ = 0.10) and reduced in ENR-CD (R^2^ = 0.33). *n* = 36 (ENR), 49 (ENR-CV), and 37 (ENR-CD) from two independent experiments (S4 Data). (E) Number of branches was measured over 4 d of organoid growth and increases over time. No difference was detected in the number of branches between organoids grown in ENR and ENR-CV (*p* > 0.05 at all time points, *t* test). Organoids grown in ENR-CD had a significantly reduced number of branches from 30 h (*p* > 0.05 until 24 h, *p* < 0.05 from 30–96 h, *t* test). *n* = 40 organoids from four replicates for ENR; 35 organoids from four replicates for ENR-CV; and 19 organoids from three replicates for ENR-CD (S5 Data). Underlying data for panel D can be found in S4 Data, and for panel E in S5 Data.

The documented expansion of the crypt base [25] prior to fission suggests that the number of Paneth and/or Lgr5+ cells in a crypt increases as a crypt ages. We found a strong correlation between the length of a crypt and the number of Paneth cells it contained (Fig 6D and S9 Fig, R^2^ = 0.67, S4 Data). Comparing single and fissioning crypts suggested that organoid crypts grow longer as they age until they reach approximately 100 μm and contain approximately 12 Paneth cells (S10 Fig, S4 Data) before they fission, similar to the number of Paneth cells reported in tissue [26].

Organoids permit manipulating the cellular composition of crypts so that the requirement for different cell types in fission can be examined. Lgr5+ cell numbers can be increased by supplementing standard growth media (ENR) with Chiron99032 (C) and Valproic acid (V) (ENR-CV), while Paneth cell number can be increased by ENR-CD (standard growth media supplemented with Chiron99032 and DAPT [D]). Both growth conditions caused altered crypt morphology: crypts in ENR-CV were longer, while crypts in ENR-CD were shorter and rounder (Fig 6B–6D, S9 Fig). In both conditions, the correlation between Paneth cell number and crypt length was lost (Fig 6D, S9 Fig, and S4 Data; ENR, R^2^ = 0.67; ENR-CV, R^2^ = 0.10; and ENR-CD, R^2^ = 0.33). The fissioning of crypts in organoids grown in ENR-CV was not altered measurably (Fig 6E, S5 Data), but crypts grew longer than 100 μm before fission (S10 Fig, S4 Data). On the other hand, fission incidence was reduced in organoids grown in ENR-CD (Fig 6D, S4 Data). These data indicate that onset of fission is not related to the absolute numbers of Paneth or Lgr5+ cells per crypt, but that a combination of their relative abundance and position is important.

### Mislocalising Paneth Cells Changes the Symmetry of Fission

**Fig 7:**
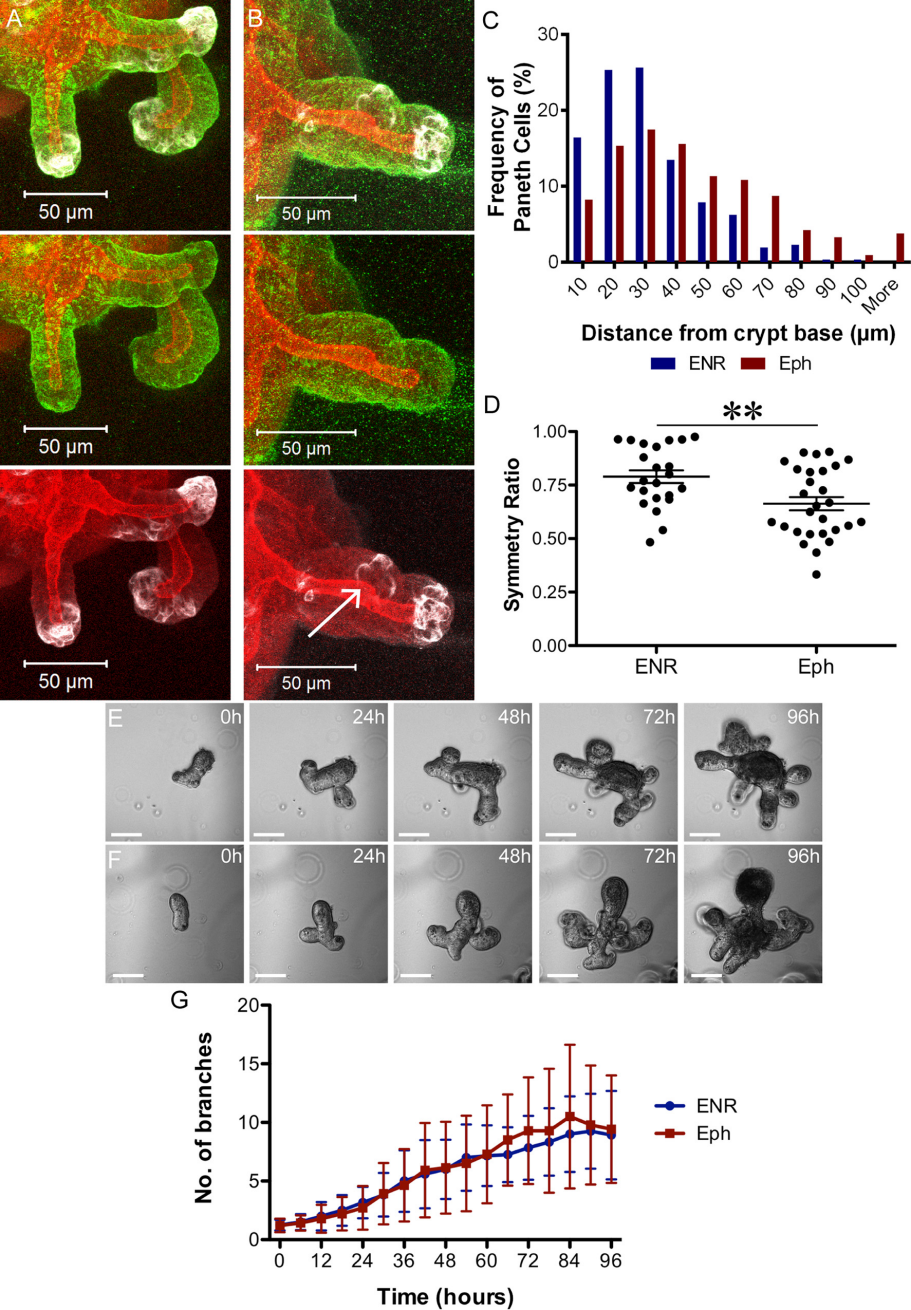
Mislocalising Paneth cells leads to more asymmetric fission. Shown are 3D projections of organoids stained to visualise F-actin (Phalloidin, red), Paneth cells (Lysozyme, white), and β4 Integrin (green), grown in control (ENR) media (A) or after the addition of an inhibitory Eph fragment (B). Note the bud formation between three Paneth cells located away from the base of the crypt (arrow in B). (C) The frequency of Paneth cells along the crypt axis was recorded relative to the crypt base and is shown in a histogram (bin every 10 μm), revealing their displacement away from the crypt base after addition of the Eph fragment (*n* = 304 Paneth cells from 48 crypts for ENR and 423 Paneth cells from 45 crypts for Eph, S6 Data). The crypt base was defined as the tip of an organoid branch. (D) Symmetry ratio (length of short daughter ÷ length of long daughter) in organoids grown in ENR with or without Eph fragments shows that organoids grown in the Eph fragment have fewer symmetrical fissions (*p* < 0.01; *n* = 23 for ENR and 28 for Eph, S7 Data). Organoids grown in the absence (E) or presence (F) of inhibitory Eph fragments were recorded by time-lapse imaging and (G, S8 Data) fission incidence, defined as number of branches over time, plotted. *N* = 14 organoids from three biological replicates; *p* > 0.05 for all time points (*t* test). Underlying data for panel C can be found in S6 Data, for panel D in S7 Data, and for panel G in S8 Data.

**Fig 8:**
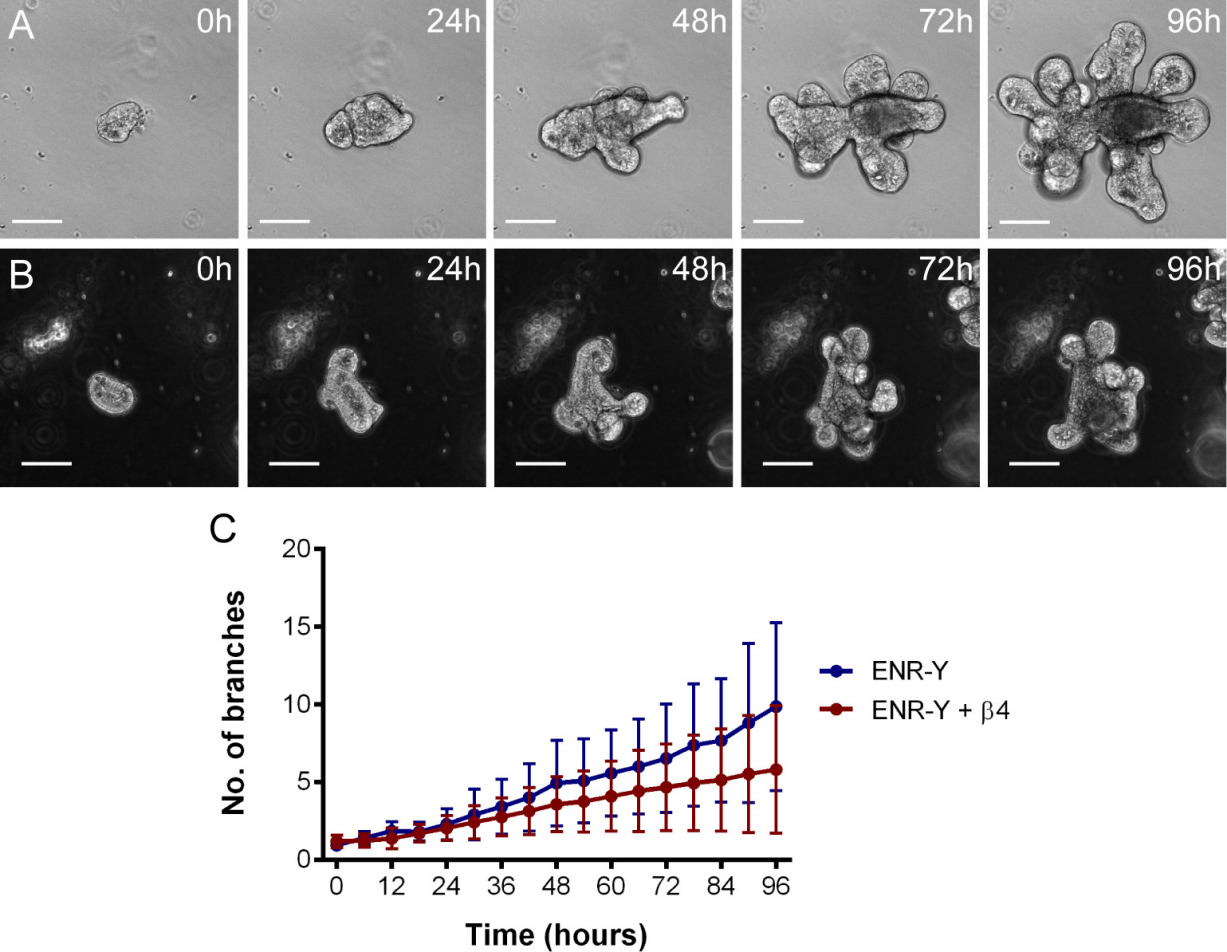
Inhibiting β4 Integrin decreases fission. Organoids grown in control media (ENR-Y; A) or in the presence of a β4 Integrin blocking antibody (B) were recorded by time-lapse imaging. The number of branches were measured every 6 h (C, S9 Data). Branch number increases more slowly in organoids grown in the presence of the β4 Integrin antibody (*p* < 0.05 from 78 h, *t* test; *n* = 21 from three replicates for both conditions), indicating a lower incidence of fission. The different background in panels A and B arises from phase contrast effects at different positions in a well relative to its centre that affect background intensity. Underlying data for panel C can be found in S9 Data.

New branches in organoids are reported to form at the location of Paneth cells; therefore, we examined whether mislocalising Paneth cells could affect crypt fission. Eph/Ephrin signalling is important for the normal positioning of Paneth cells [27], and inhibiting Eph/Ephrin signalling in mice causes mislocalisation of Paneth cells from the crypt base [28]. We found that including inhibitory Eph fragments in organoid culture media also caused mislocalisation of Paneth cells to positions above the crypt base (Fig 7A–7C, S6 Data). Fission events became more asymmetrical in the presence of the Eph fragment, indicating new branches formed further from the crypt base (Fig 7D, S7 Data). Time-lapse imaging revealed that the number of crypts increased at similar rates in Eph-treated and control organoids, indicating fission incidence was unaffected (Fig 7E–7G, S8 Data). Importantly, the Eph fragment had no effect on the increased β4 Integrin signal intensity on the basal surface of Paneth cells (S11 Fig, S2 Data). If the increased adhesion of Paneth cells is important for their role in positioning fission, we would expect inhibiting cell-substrate adhesion to reduce fission. Indeed, organoids grown in the presence of a β4 Integrin blocking antibody showed reduced incidence of fission compared to controls (ENR + Y27632; Fig 8, S9 Data). Together, these data suggest that Paneth cells are important in positioning daughter crypts and that the differential adhesion of Paneth cells and their neighbours is involved in normal fission. This supports the idea that differential adhesion of Paneth and Lgr5+ cells is important in positioning the buckling event that initiates fission.

### Mathematical Modelling Supports Observations that Both Paneth and Lgr5+ Cells Are Required for Crypt Fission

**Fig 9:**
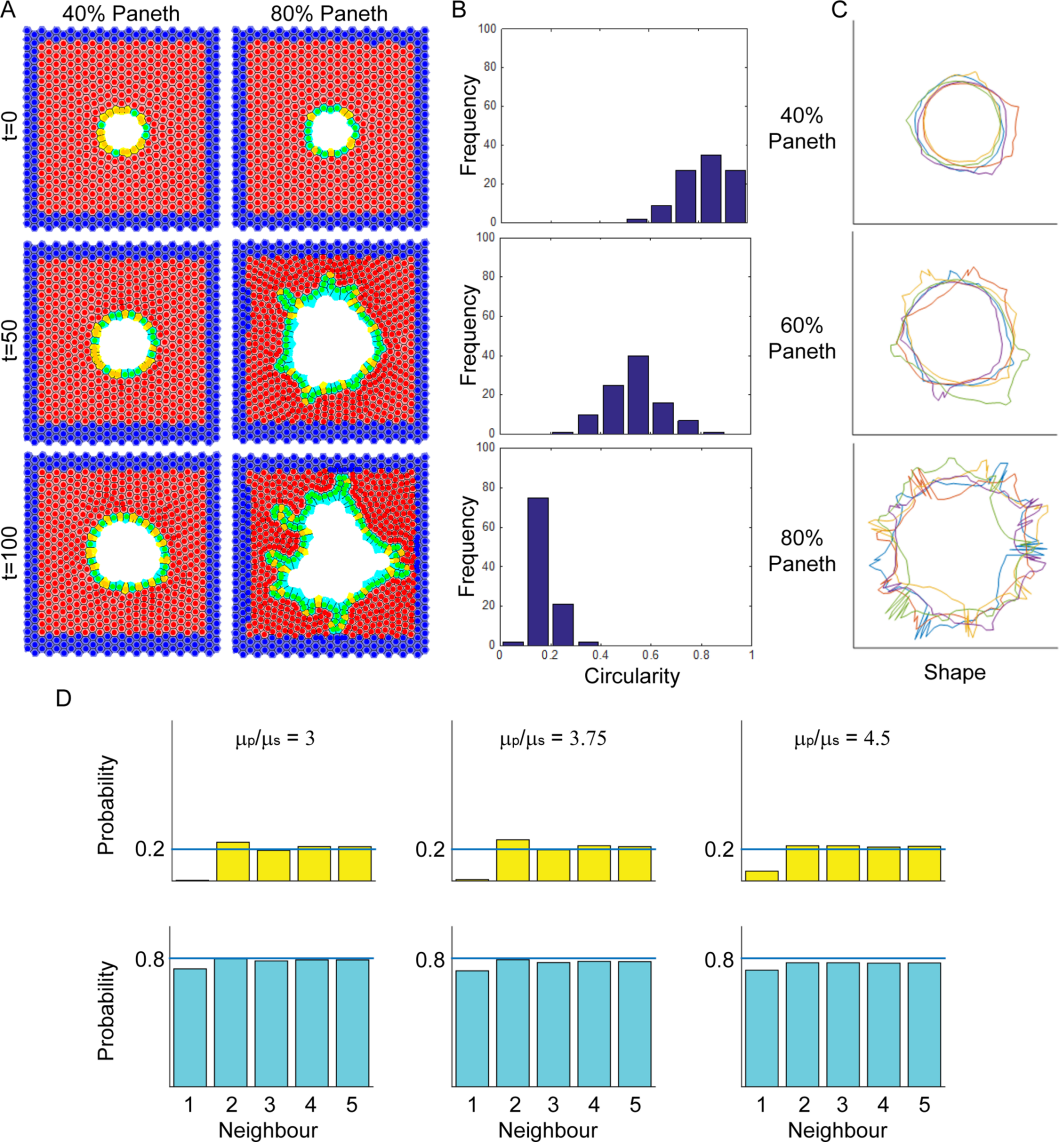
Mathematical modelling indicates Paneth cells stimulate deformation. (A) Images from a sample simulation of fission in the epithelial layer at times t = 0, 50, and 100 h. A high proportion of stiffer Paneth cells (light blue) causes buckling of the epithelial layer. Softer Lgr5+ cells (yellow) are pushed out of the initially spherical layer by neighbouring Paneth cells, leading to the formation of a bud. At later times and higher proportions, Lgr5+ cells also appear in bifurcation sites between small buds (bottom right panel). Red cells represent Matrigel or surrounding stromal cells; blue cells represent non-epithelial cells fixed around the edges. (B) Distributions of the final circularity values of the epithelial layer for different Paneth cell proportions (S11 Data). Each histogram has been plotted with ten evenly spaced bins from 0 to 1. Circularity values closer to one reflect a circular layer, while values closer to zero reflect a less regular circle, which indicates buckling of the cell layer. (C) Five representative portraits of the epithelial layers for different Paneth cell proportions. (D) Bar graphs displaying probability of a neighbouring cell being of the same type (S12 Data). Top panel shows probability of a stem cell occupying each of the five neighbouring cells to a stem cell (at position 0). Lower panel shows the same for Paneth cells. Only in cell layers where Paneth cells are 4.5-fold stiffer (μ_p_/μ_s_ = 4.5) and crypt wall deformation occurs do two stem cells neighbour each other, apparent by neighbour 1 having a probability value >0. Underlying data for panel B can be found in S11 Data, and for panel D in S12 Data.

Experimental data from organoids suggests a role for both Paneth and Lgr5+ cells during fission. However, our data that increasing the number of Paneth cells reduced fission contradicts our observation that new branches form where Paneth cells are located. To examine this in more detail, we utilised computational modelling of connected epithelial layers to investigate the role of Paneth cells in fission. Paneth cells were modelled to be 4.5 times stiffer than neighbouring cells [12], but they were otherwise identical to other cells in the layer. One consequence of these parameters was that Lgr5+ cells were smaller than Paneth cells (S12 Fig, S10 Data). Paneth cells were 1.4-fold or 1.14-fold larger than Lgr5+ cells for epithelial layers containing 40% or 80% Paneth cells, respectively. This effect is more marked when cells are in a constrained stable circular layer (as seen at 40% Paneth cells), as in a highly constrained system the cell stiffness has a greater influence on cell size and shape (S12A Fig). We observed more buds forming in epithelial cell layers containing a higher proportion of Paneth cells (Fig 9A). The mechanism of budding initiation was similar to that reported by Pin et al. [12], in which an Lgr5+ cell is pushed out by surrounding Paneth cells and proliferates, resulting in bud formation. Circularity of the epithelial layer was used as a measure of deformation. Epithelial layers with fewer Paneth cells had circularity values distributed around one (Fig 9B, S11 Data), indicating a circular shape. Increasing the proportion of Paneth cells led to decreased circularity with values tending toward zero (as demonstrated in Fig 9C), indicating that the epithelial layer had deformed. These data support a model in which Paneth cells are required to initiate outward buckling, mimicking a budding event. This appears to be contradictory to our observation that increasing Paneth cell numbers reduces fission (Fig 6). It is likely that this is due to the lack of Lgr5+ cell clustering under these conditions. Indeed, when we examined how the ratio of cell stiffness influenced fission, we found (for epithelial layers containing 80% Paneth cells) a similar progression from stability to fission as we increased the stiffness ratio from 3 to 4.5. Importantly, after budding is initiated, two Lgr5+ cells adjacent to the budding site are more likely to neighbour each other (Fig 9D, S12 Data). This is reminiscent of the Lgr5+ cluster that we found experimentally in early fission. Together with experimental data, these findings suggest that Paneth cells are required to deform the crypt base, while Lgr5+ cells may expand the region between Paneth cells to separate the new prospective crypt bases. In organoids, the region between Paneth cells is expanded by cell division [12]. Consistently, we observed mitotic cells in the bifurcation in fissioning crypts in tissue (S13 Fig), indicating that mitosis may play a role in expanding the Lgr5+ cell cluster in tissue.

## Discussion

Crypt fission is at the core of normal growth and maintenance of intestinal tissue. Importantly, the normal decrease in fission that occurs with ageing is reversed in early cancer, and aberrant fission in adult tissue is associated with adenoma growth. This study aims to determine how fission works normally so that mechanisms involved in fission in adenoma growth can be identified more readily. We found that the initiation of fission was marked by a cluster of Lgr5+ cells in the middle of the crypt base. Paneth cells appeared only lateral to this region and marked the bases of prospective daughter crypts. We discovered that differential adhesion of Paneth and neighbouring Lgr5+ cells to the basement membrane via β4 Integrin is likely involved in fission. This conclusion was supported by our finding that blocking β4 Integrin inhibits fission in intestinal organoids.

### Asymmetric and Triple Fission Are Normal Events in Development

To identify how fission changes in cancer, we first had to establish what constitutes normal fission in situ. We found symmetric and asymmetric fission with similar frequency in healthy tissue, contrary to a recent report that identified “asymmetric budding” as the prevalent form of crypt fission [16]. These differences are easily accounted for by different definitions of asymmetric fission. We classified asymmetric fissions as those producing daughter crypts varying by ≥25% in length compared to >1% [16]. We also found that triple fission is a normal event and is not restricted to adenoma. However, it was only detectable during early postnatal development. It is possible that triple fission is a feature of rapidly growing tissue, when the space available due to lower crypt density allows triple fissions to occur more readily. Indeed, higher fission rates after irradiation have been reported to be a result of lower crypt densities [2]. Similarly, the high fission incidence in early postnatal development observed here may reflect lower crypt densities. We report that fission incidence reduces most rapidly in the distal regions of both the small intestine and colon, which may be explained by the different crypt morphologies [29] or different signalling pathway activities (e.g., Wnt, [30,31]) along the proximal-distal axis.

### Initiation of Fission Involves a Specific Arrangement of Paneth Cells and Lgr5+ Cells

Fission incidence in different regions of the gut correlates with mitotic rate [32]. However, simply increasing mitotic rate does not stimulate crypt fission [33]. Instead, we identified changes in cell patterning, specifically the appearance of a cluster of Lgr5+ cells between at least two Paneth-cell-rich areas to establish regions of different mechanical properties at the crypt base.

One question that remains is how the Lgr5+ cluster forms. Our computer model provided some clues. First, it showed that the greater stiffness of Paneth cells deforms the crypt wall (Fig 9), consistent with our finding that Paneth cells determine where fission occurs (Fig 7 and [12]) and suggesting that Paneth cells may determine the shape of the crypt base. Second, the model showed that in the vicinity of the deformation caused by the Paneth cells, Lgr5+ cells are more likely to neighbour each other (Fig 9). The predicted increased probability of two Lgr5+ neighbours near deformations is consistent with our finding of Lgr5+ cell clusters at these sites. Together this suggests that the Lgr5+ cell cluster may be a consequence of crypt wall deformation initiated by Paneth cells. Alternatively, or in addition, the differences in Lgr5+ and Paneth cell behaviour may lead to Lgr5+ cell cluster formation. Lgr5+ cells divide [21], while Paneth cells are generated slowly [34] and reside at the crypt base for 18–23 days [35,36]. These data and our findings that Paneth cells are more adherent suggest that Paneth cells lack mobility. Together, this suggests that Lgr5+ cells produced at the extreme crypt base may be trapped by their larger, stiffer, and less mobile neighbours, the Paneth cells, causing Lgr5+ cells to cluster. This idea is further supported by the recent report that displacement of stem cells from the extreme crypt base is less likely than displacement from more lateral positions bordering the niche [37].

Once the Lgr5+ cluster has formed, it creates a softer area in the crypt base. This area deforms in response to forces exerted by neighbouring crypts and results in the upward buckling event that initiates the formation of new crypt walls. Expansion of these walls is likely achieved by proliferation of the Lgr5+ cells in the cluster, as suggested by the mitotic cells we observed in the bifurcation of fissioning crypts (S13 Fig). One consequence of Lgr5+ clustering is a loss of their direct contact with Paneth cells. This is predicted to cause Lgr5+ cells to become transit-amplifying cells [37], as indicated by the loss of Lgr5-GFP expression in bifurcations extending beyond the stem cell compartment (Fig 3). When there are too many Paneth cells, Lgr5+ cells may not be able to form a cluster, explaining the reduced incidence of fission in organoids grown in ENR-CD (Fig 6).

### Genetic Evidence that Paneth Cells Are Required for Crypt Fission

An important role for Paneth cells in normal fission is supported by many other reports: organoids lacking Paneth cells do not form branches [13,14,38]; high incidence of fission in tissue in young mice coincides with a rapid increase in the number of immature Paneth cells per crypt [39–41]; and *Sox9*^*flox/flox*^ mice, which are depleted of Paneth cells, have wider and deeper crypts [42,43], which could reflect a failure to fission.

Apparently inconsistent with our conclusions that Paneth cells are important for crypt fission are studies suggesting they are not required for recovery after injury. In Paneth-cell-depleted mice (*CR2-tox176*, *Math1*^*flox/flox*^ and *Gfi1*^*KI/KI*^ [14,15,44–47]), intestinal tissue can recover after radiation-induced injury. However, 10Gy ^137^Cs irradiation [14,15] does not induce severe crypt loss [15,48,49], and recovery may not require crypt fission. Alternatively, other secretory cells may substitute for Paneth cells. Indeed, the UEA-I+ and Muc2+ cells still present in *Gfi*^*KI/KI*^ mice [44,46,47] also had an increased signal intensity of β4 Integrin on their basal surface compared to their neighbours (S6 Fig), a feature we found to be important for fission.

### Increased Substrate Adhesion of Paneth Cells Is Important for Normal Fission

Increased adhesion of Paneth cells to the underlying basement membrane (Fig 4) is consistent with increased β4 Integrin on their basal surfaces [50–54]. Inhibiting β4 Integrin causes reduced fission incidence in organoids, consistent with a role of cell-substrate adhesion in fission (Fig 8). Although our model does not take substrate adhesion into account and only incorporates the increased stiffness of Paneth cells, it still produces results consistent with our experiments showing that Paneth cells drive the budding process required for crypt formation in organoids. The higher stiffness of Paneth cells may be sufficient to drive fission, while their increased adhesion may make the process more efficient in normal tissue by anchoring the crypt bases. *Apc* mutant organoids have a uniform distribution of β4 Integrin on their basal surface and fail to form branches (S4 Fig; [55]), suggesting that differential adhesion between the different cell types in the crypt is important for fission.

Understanding how the differential accumulation of β4 Integrin in secretory cells is achieved, and how it changes in polyps, requires identifying factors that regulate its expression. One potential factor is Wnt, a key regulator of intestinal crypt homeostasis [56]. For instance, the Wnt-responsive transcription factor c-Myc can regulate activity of the *Itgb4* promoter [57–59].

Growth of many other tissues also involves branching structures that bear some similarities to fissioning crypts and also involve differential adhesion; for instance, milk duct formation in the breast. However, there are also distinct differences between these cases. Crypt fission in the intestine and colon initiates from a stationary crypt base that is relatively fixed in space by the surrounding tissue layer. Mammary ducts achieve branching by growth of two terminal end buds into extracellular space with a stationary, non-growing area between. Weaker substrate adhesion has been reported at the terminal end buds of breast tissue [60]. Therefore, similar to crypt fission, substrate adhesion is weaker in areas where tissue actively changes shape and moves. Another similarity is that β1 and β4 Integrin are important regulators of breast development and may play a role in breast tumour progression [61]: β4 overexpression correlates with more rapid breast cancer progression [62] and is required for ErbB2-driven tumorigenesis [63]. As a consequence, integrins have been identified as potential targets for treatment of breast cancer [61]. Any antagonists developed may provide useful tools for investigating the role of β4 Integrin in colorectal cancer and may lead to new avenues for treatment.

### A Model for Crypt Fission

**Fig 10:**
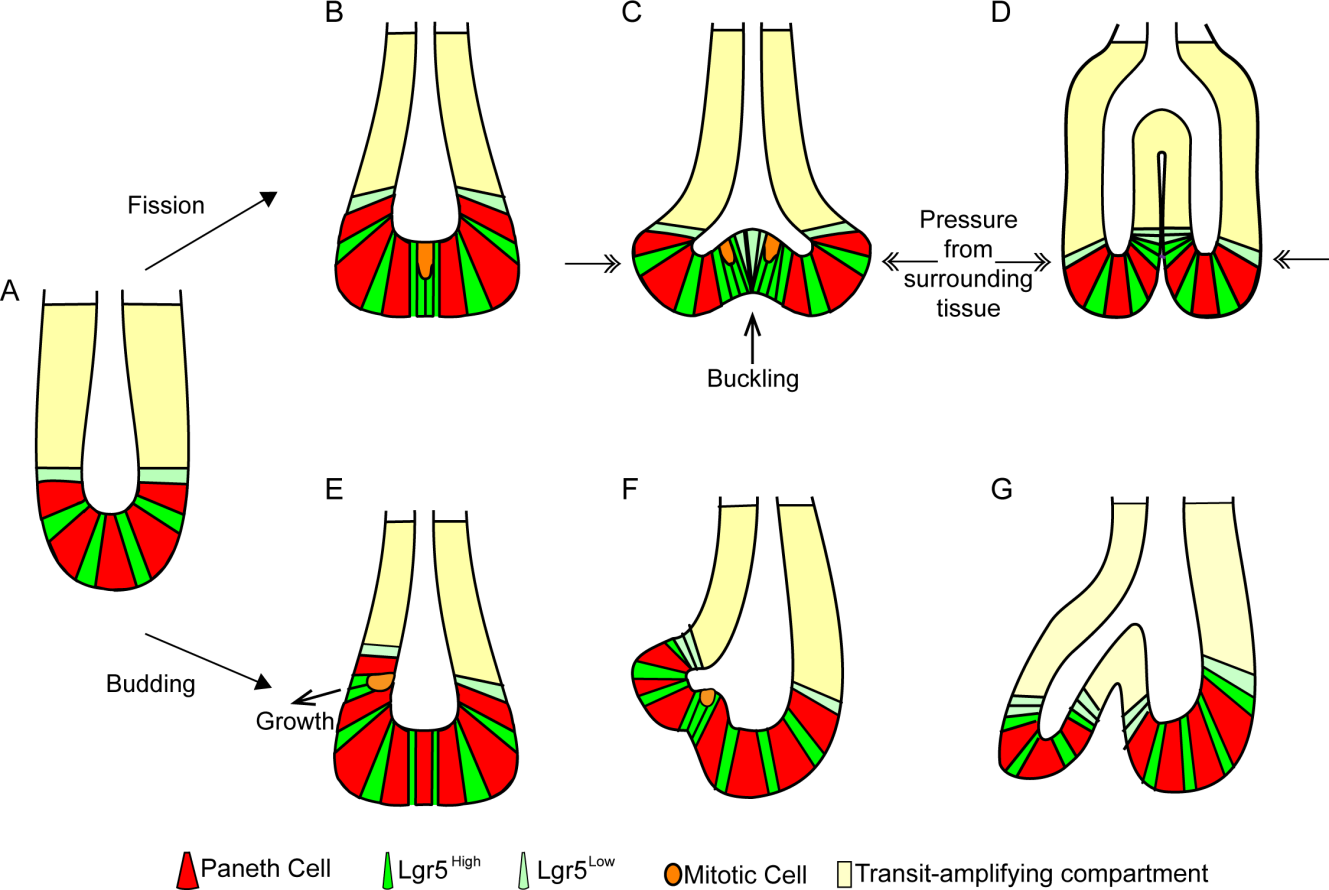
Summary of the mechanisms involved in crypt fission and budding. The stem cell niche of a single intestinal crypt (A) is composed of alternating Paneth and Lgr5+ cells. Over time, crypts become larger, and expansion of the crypt base, together with Lgr5+ cell division, leads to formation of clusters of Lgr5+ cells (B). The softer Lgr5+ cells are likely more prone to buckling (C), leading to bifurcation of the parental crypt. (D) Expansion of the buckled region leads to the formation of new crypt walls and, eventually, two daughter crypts. Pressure from surrounding tissue (double-headed arrows) may contribute to buckling and keep the two daughter crypts parallel to each other. Similar to fission, budding requires an expansion in the number of Lgr5+ cells between two Paneth cells (E). The crypt wall buckles outward and gradually expands over time (E–G) to create a smaller daughter crypt branching off the side of the parental crypt.

We report here mechanisms involved in intestinal crypt fission (summarised in Fig 10). Crypt fission involves a specific arrangement of cells within the intestinal stem cell niche. Lgr5+ cells cluster between Paneth cells, possibly as a result of mitotic daughters at the extreme crypt base becoming trapped between neighbouring Paneth cells. The Lgr5+ cell cluster creates a softer region that is more prone to changing shape in response to mechanical pressure from surrounding tissue. The Lgr5+ cell cluster expands upward from the middle of the crypt base, causing a bifurcation of the parental crypt as described by Wright [64]. The presence of mitotic cells in this region (S13 Fig) suggests that proliferation may play a role in the expanding bifurcation. The related process of crypt budding, which occurs above the crypt base, involves the proliferation of a cell positioned between two Paneth cells as reported by Pin et al. (Fig 10) [12]. The new bud grows through proliferation of cells positioned between the Paneth cells. In both fission and budding, proliferation is likely to support expansion of new crypts. While fission (either symmetric or asymmetric) is the prevalent form of crypt formation in tissue, new crypts form in organoids through both fission and budding-like branching. In both fission and budding, the position of Paneth cells dictates the site of fission. Their higher stiffness and adhesion helps to anchor and shape the new crypt bases.

### Conclusion

Crypt fission is important for growth of the gut and plays a role in recovery from injury, but it is also responsible for expansion of mutant tissue in polyps and adenoma. Here, we describe how fission occurs normally. Our findings suggest that regulation of β4 Integrin, particularly at the crypt base, is involved. Mis-regulation of Integrins in colorectal cancer development may contribute to the formation of aberrant crypt fissions common in adenomas. Understanding regulation of β4 Integrin in normal and early cancerous tissue may reveal how crypt fission increases during adenoma formation. Further examination of the role of β4 Integrin and cellular adhesion in crypt fission requires the development of novel mouse models, allowing the conditional depletion of adhesion molecules in the intestine. Potential similarities between the role of Integrins in colorectal and breast cancer may make drugs that target Integrins useful for treatment of both of these common human cancers.

## Material and Methods

### Ethics Statement

All experiments involving animals were performed in accordance with United Kingdom Home Office approved guidelines and were approved by the Home Office Licensing Committee (project license PPL60/4172).

The Tayside Tissuebank subcommittee of the Local Research Ethics Committee approved the collection of tissue samples. Normal human samples were obtained from surgical resections for hemi-colectomy.

### Preparation and Staining of 3D Whole Mount Tissue

All experiments involving animals were performed under the UK Home Office guidelines. CL57BL/6 wild-type, *Lgr5-EGFP-IRES-creERT2* (*Lgr5*^*GFP/+*^), and *Apc*^*Min/+*^ were sacrificed by cervical dislocation or exposure to CO_2_, and the entire intestine (small and large) was removed immediately. Tissue was washed and divided into proximal, medial, and distal regions of the small and large intestines using percentage length from the gastro-duodenal and ileo-caecal junctions [8] corresponding to six regions. Regions for the small intestine were: region 1, 10% = duodenum; region 2, 50% = jejunum; region 3, 90% = ileum. Regions for the large intestine were: region 4, 10% = caecum; region 5, 50% = medial; region 6, 90% = distal. A 5 mm x 5 mm section from the middle of each of these six regions was immersed in cold fixative containing 4% paraformaldehyde in PBS (pH 7.4) overnight at 4°C before processing for staining [17].

Tissue samples were prepared for optical imaging of F-actin and nuclei as described previously [17]. For staining of Lgr5^GFP^+ cells and Paneth cells, an additional antibody incubation step and additional washing was added. Lgr5^GFP^+ cells were visualised using anti-GFP (Abcam, Cambridge, UK) and Paneth cells with Lysozyme (1:2,000, Dako, Cambridge, UK). Secondary antibodies (AlexaFluor-conjugated 1:250, Molecular Probes, Eugene, OR) were added along with fluorescently labelled Phalloidin (AlexaFluor-conjugated 0.26 μM [8 units], Molecular Probes) and Hoechst (50 μg/ml, Molecular Probes). Specimens were mounted in BABB after dehydration through an ethanol series [17].

### Organoid Culture

Organoids were generated from mouse small intestinal crypts as described previously [13]. Briefly, small intestine was removed from the mouse and flushed. The intestine was opened longitudinally and villi removed by scraping the luminal surface with a coverslip. Tissue was washed with PBS, incubated in 3 mM EDTA (20 min), and crypts detached mechanically by vigorous shaking. Crypt suspension was washed twice in PBS then once in Advanced DMEM/F12. Crypts were dissociated to single cells with TrypLE Express (Life Technologies, Carlsbad, CA) at 37°C for 5 min; Advanced DMEM/F12 (Life Technologies) was added, then cells were filtered through a 40 μm cell strainer (Greiner, Frickenhausen, Germany). Single cells were then suspended in Growth Factor Reduced Phenol red free Matrigel (BD Biosciences, Oxford, UK). Organoids were grown in crypt media (Advanced DMEM/F12 supplemented with 10 mM HEPES, 2 mM Glutamax, 1 mM N-Acetylcysteine, N2 [Gemini, Sacramento, CA], B27 [Life Technologies], Pen/Strep [Sigma-Aldrich, St. Louis, MO]) containing growth factors (EGF [50 ng/ml; Invitrogen], Noggin [100 ng/ml; eBioscience], and R-Spondin conditioned media [1:4]). Additional growth factors Chiron99021 (3 μm; Invitrogen, Waltham, MA), Valproic acid (1 mM; Invitrogen), and Y27632 (10 μm; Cambridge Bioscience, Cambridge, UK) were added for the first 48 h culture. Organoids were passaged by first physically breaking up Matrigel, then washed in Advanced DMEM/F12 and dissociated into individual crypts by pipetting. Individual crypts were re-suspended in Matrigel and grown in crypt media containing growth factors.

Additional reagents were added along with the crypt media. Chiron99021, Valproic acid, and Y27632 were added at the same concentrations as described above. 1 μg/ml recombinant mouse EphB2 Fc chimera (R&D Systems, Minneapolis, MN) was added at concentrations corresponding to serum values previously reported [28]. Anti-β4 Integrin (Abcam) was added at 5 μg/ml. In the presence of a β4 Integrin antibody, organoids become moribund and disintegrate after 2 d. However, including the Rho kinase inhibitor Y27632 to inhibit anoikis [13] prevents this demise, confirming that adhesion to a basement membrane is compromised by the β4 Integrin antibody.

### Immunofluorescent Staining of Organoids

Organoids were grown in Matrigel in 8-chamber μ slides (Ibidi, Munich, Germany) for 3–5 d and fixed in warmed 4% paraformaldehyde in PBS (pH 7.4) for 20 min (37°C), permeabilised for 1 h in 1% Triton-X100 (this and all subsequent steps were carried out at RT), and blocked for 1 h (1% BSA, 3% normal goat serum, 0.2% Triton-X100 in PBS). Organoids were incubated in antibodies overnight in Working Buffer (0.1% BSA, 0.3% normal goat serum, 0.2% Triton-X100 in PBS): β4 Integrin (1:100 Abcam), Lysozyme (1:2,000), washed 5x in Working Buffer, then incubated in secondary antibodies (1:250, AlexaFluor-conjugated [Molecular Probes]), along with 5μg/ml Hoechst 33342 (as above) and Phalloidin (as above) overnight in Working Buffer, washed 5x in Working Buffer, and mounted in ProLong Gold antifade (Molecular Probes).

### Preparation and Staining of Sectioned Tissue

Small intestine and colon were isolated from mice and flushed with PBS followed by 4% paraformaldehyde (pH 7.4). Tissue was opened longitudinally, washed briefly in PBS, and incubated in 4% paraformaldehyde (pH 7.4) at 4°C overnight. A small, square piece of tissue was excised with a scalpel, embedded in 3% Low Melt Temperature Agarose, and sliced into 200 μm thick sections with a Vibratome (Leica, Wetzlar, Germany). Tissue sections were permeabilised for 2 h in 2% Triton-X100 (this and all subsequent steps at 4°C), blocked for 2 h (1% BSA, 3% normal goat serum, 0.2% Triton-X100 in PBS), and incubated in primary antibodies for 3 d (Lysozyme and β4 Integrin as described above, Muc2 [1:200, Santa Cruz Biotech, Santa Cruz, CA]), washed in Working Buffer (5x in 1 h), incubated in secondary antibodies along with Phalloidin and Hoechst (as described above) and Rhodamine-labelled UEA-I (1:200, Vector Labs, Peterborough, UK), washed in Working Buffer (5x in 1 h), and mounted in ProLong Gold antifade (Molecular Probes). Sections were mounted on coverslips between 2x 120 μm spacers to preserve tissue structure.

### Microscopy, Image Processing, and Analysis

Tissue and organoids were imaged with a Zeiss LSM 710 microscope (Carl Zeiss AG, Oberkochen, Germany) using 25x or 40x Zeiss objective lenses and immersion oil with a refractive index of 1.516. Multiphoton excitation was provided by a Coherent Chameleon Titanium Sapphire laser at 820 nm to simultaneously excite Hoechst and Rhodamine-Phalloidin for whole mount tissue imaging. Organoids and sectioned tissue were imaged in conventional confocal mode using 40x LD Plan-Neofluar objective lens, and Z stacks were taken at 1 μm steps.

Image processing and analysis were performed in Volocity (PerkinElmer, Waltham, MA) or Imaris (Bitplane, Windsor, CT). Fission in tissue was measured by importing image stacks of 3D whole mount tissue into Volocity. Using the images, all crypts were marked and those undergoing fission identified to report the proportion of all crypts undergoing fission. Symmetry and fission stage was determined visually: using a cut-off of approximately 25% divergence in daughter crypt lengths and sizes to mark an asymmetric fission. The structure of fission was determined in Imaris: surfaces were drawn around the crypt lumen and outer wall based on Phalloidin staining. Using Imaris, crypt length was determined by measuring the position of the crypt base and the crypt opening, while symmetry was assessed by dividing the length of the shorter daughter crypt by the length of the longer daughter crypt. Each Paneth cell was marked and its distance from the crypt base measured to analyse number and distribution of Paneth cells. β4 Integrin levels on basal cell membranes were measured using Imaris; surfaces were drawn around the basal membrane of Paneth cells and their neighbouring cells. Average signal intensity in these surfaces was measured for each individual cell. The intensity for β4 Integrin in Paneth cells was divided by the corresponding value for its neighbours.

### A Mathematical Model of Fission in a Connected Epithelial Layer

We extend the crypt model outlined in Dunn et al. [65] to model the cross-section of a confluent epithelial layer. We represent cells by the positions of their centres and define spatial connectivity through a Delaunay triangulation. Cells interact with each other through forces applied along the edges of the triangulations. Attraction and repulsion between cells is modelled through a linear spring force law. Furthermore, epithelial cells are subjected to a basement membrane force, where the basement membrane is defined along the boundary separating epithelial cells and the Matrigel. Detailed mathematical descriptions of these forces and simulation details are provided in Dunn et al. [65,66]. The code used to run these simulations can be found at https://chaste.cs.ox.ac.uk/trac/wiki/PaperTutorials/CryptFissionPlos2016.

We consider a 20-by-20 square array of cells, consisting of a layer of proliferative epithelial cells surrounded by terminally differentiated non-epithelial cells (representing the surrounding stromal cells or Matrigel). Each simulation begins with a circular epithelial layer mimicking the base of the crypt (i.e., two bases joined together, or the initial geometry of spherical organoids [13]). The region surrounded by the epithelial layer represents the crypt lumen. Outside the square, additional layers of non-epithelial cells are fixed around the edges, ensuring the layer may deform without compromising the overall shape of the box. Any cell that moves beyond any of the four walls of the square also becomes fixed.

The epithelial layer consists of both Lgr5+ and Paneth cells. Epithelial cells proliferate according to a stochastic cell cycle model, in which the cell cycle duration is sampled from a Uniform Distribution *U(12*,*14)* [67]. Each cell can divide symmetrically or asymmetrically according to a pre-defined division parameter controlling the proportion of stem cells in the model, called the target proportion. We model Paneth cells as stiffer than Lgr5+ cells, by assigning the stiffness of springs connecting one or more Paneth cells to be 4.5 times stiffer [12] than springs joining only Lgr5+ cells or non-epithelial cells (whose stiffness is as defined in [64]). Apart from stiffness, Paneth cells are physically identical to stem cells in simulations. Note that we are considering the cross-section of a three-dimensional epithelial layer and modelled all epithelial cells to have the same size. Normally, Paneth cells are 2–4-fold larger than Lgr5+ cells. As a consequence, the model requires a larger number of Paneth cells than normally present in tissue. To prevent over-crowding in the layer, cell death in the form of anoikis is introduced. If an epithelial cell loses contact with the surrounding non-epithelial cells and is pushed into the lumen, it is removed from the simulation.

For each target proportion value, 100 simulations were run for 100 h each. This length of time was in accordance with the length of time taken for organoids to undergo budding [13]. To determine incidence of crypt fission, we track the circularity of the epithelial layer, defined by the formula $C=\frac{4\pi ∙Area}{({Perimeter)}^{2}}$. More circular forms have circularity values closer to one, while more complex shapes will have circularity values near zero. An epithelial layer with low circularity indicates crypt fission has occurred. As a buckled epithelial layer cannot revert back to a circular shape, we need only to record the circularity at t = 100 h.

### Live Imaging of Organoid Cultures

Live imaging of organoids was performed using confocal microscopy, as described above, for LifeAct-GFP organoids (kind gift from Prof. Laura Machesky, Beatson Insitute, Glasgow, UK). Brightfield movies were recorded with a Leica DMIRB (Leica) inverted microscope and analysed in Fiji [68]. The number of branches per organoid was counted every 6 h using the Cell Counter plugin. Numbers of branches shown on graphs correspond to the average number of branches per organoid.

### Adhesion Assays

Single cells were isolated from mouse small intestinal crypts, as described above for organoid culture. Cells were plated in Advanced DMEM/F12 on laminin-coated (Sigma-Aldrich) surfaces and allowed to adhere for 1 h. Cells were shaken at 2,000 rpm for 15 s to remove weakly adherent cells. Cells were fixed in 4% paraformaldehyde, and stained with Hoechst and Lysozyme (as described above). The total number of cells and Paneth cells were counted and compared to non-shaken controls.

### Statistical Analysis

All statistical analyses were performed using GraphPad Prism 6.0a (GraphPad, La Jolla, CA) for Windows. Tests performed are described in individual Figure legends, along with *p*-values and significance (ns = not significant, * = *p* < 0.05, ** = *p* < 0.01, *** = *p* < 0.001, **** = *p* < 0.0001).

## Supporting Information

S1 Data Incidence of crypt fission during murine postnatal development. Fission incidence was counted in the intestine during early postnatal development. The number of fission events for each type of fission was counted for each region. Total fission incidence was determined by adding each type together and compared to the total number of crypts analysed. Fission incidence for individual mice are shown, as well as the average (percentage) values and standard deviations.
(XLSX)

S2 Data Signal intensity of β4 Integrin on the basal surface of Paneth cells and neighbouring cells. β4 Integrin signal intensity on the basal membrane was measured for Paneth cells and their neighbouring cells. The raw values are shown underneath “PC” and “Neighbour” headings, respectively. “PC/CBC” is the ratio calculated by dividing the value for the Paneth cell by that of its neighbours.
(XLSX)

S3 Data Adhesion assays. The number of cells and Paneth cells were counted in control samples (labelled “Plated”) and after weakly adherent cells were removed by shaking (labelled “Adherent”). Percentage of Paneth cells was calculated from the number of Paneth cells in each sample compared to the total number of cells. Statistical significance was assessed by a paired *t* test.
(XLSX)

S4 Data Comparison of crypt length with the number of Paneth cells. The length of each crypt was measured and the number of Paneth cells in each crypt was counted. Measurements for ENR, ENR-CV, and ENR-CD are displayed as labelled. Correlation between Paneth cell number and crypt length was determined using linear regression statistics. Each crypt was scored for branching. This scoring was used to separate single and branching crypts for the analysis in S10 Fig.
(XLSX)

S5 Data Number of branches per organoid over time. The numbers of branches of organoids were counted at 6 h time intervals from time-lapse imaging. Each column represents the number of branches in one organoid. *t* tests were performed to assess statistical significance at each time point between ENR and ENR-CV and between ENR and ENR-CD.
(XLSX)

S6 Data Distance of Paneth cells from the crypt base. Paneth cells and the crypt base were marked using the Spot tool in Imaris, and the distance of each Paneth cell from the crypt base was measured. Histogram analysis, with a bin every 10 μm, determined the distribution of Paneth cells in control organoids and organoids treated with the Eph fragment.
(XLSX)

S7 Data Analysis of fission symmetry in organoids. Length of the two daughter crypts was measured using the Measurement Point tool in Imaris. Symmetry ratio was determined by dividing the length of the shorter daughter by the length of the longer daughter.
(XLSX)

S8 Data Number of branches per organoid in Eph-treated organoids over time. The numbers of branches in organoids were counted at 6 h time intervals from time-lapse imaging for control organoids and organoids treated with the Eph fragment. Each column represents the number of branches in one organoid. *t* tests were performed to assess statistical significance at each individual time point.
(XLSX)

S9 Data Number of branches in β4 Integrin-inhibited organoids over time. The numbers of branches in organoids were counted at 6 h time intervals from time-lapse imaging for control organoids and organoids treated with a β4 Integrin blocking antibody. Each column represents the number of branches in one organoid. *t* tests were performed to assess statistical significance at each individual time point.
(XLSX)

S10 Data Relative sizes of Paneth and Lgr5+ cells. The area of stem cells and Paneth cells (1 unit = 100 μm^2^) were recorded at the end of each simulation. Size ratio was determined by dividing Paneth cell area by stem cell area. The values obtained from simulations from epithelial layers containing 40%, 60%, and 80% Paneth cells are shown as labelled.
(XLSX)

S11 Data Circularity of connected epithelial layers. Circularity of the epithelial layers was measured at the end of each 100 h simulation as described in Material and Methods. Values obtained from epithelial layers containing 40%, 60% and 80% Paneth cells are displayed as labelled. Each measurement represents one individual simulation.
(XLSX)

S12 Data Probability of neighbouring cells being the same type. For each individual cell in an epithelial layer, the cell types of the five neighbouring cells on the right-hand side were recorded. A stem cell is recorded as “0” and a Paneth cell recorded as “1.” The different sheets display values for epithelial layers with Stiffness Ratios 3, 3.75, and 4.5, as labelled.
(XLSX)

S1 Fig Asymmetric triple fission. Crypts were prepared as in Fig 1 and show that in asymmetric triple fission, the crypt lumen trifurcates into three daughter crypts with at least one of different length. (A, B) 3D projections of Hoechst (cyan) and Phalloidin (red) stained tissue from the top (A) and side (B). (A′, B′) Imaris-rendered surfaces of the same crypt. In this example, one of the daughter crypts is shorter than the other two.
(TIF)

S2 Fig Orientation of fission is not uniform. (A) Phalloidin stained small intestinal 3D whole mount tissue shows the lumens of crypts. (B) Highlighting crypt orientation by connecting the bases of daughter crypts shown in (A) reveals the diverse orientation of fissions. (C) Close-up and (D) side view of two adjacent crypts shows fissioning in different orientations (region highlighted by white box in [A]).
(TIF)

S3 Fig Fission in human tissue. Fixed human tissue stained against Hoechst (blue), Lysozyme (red), and Phalloidin (green). Paneth cells are excluded from the region underneath the bifurcation in fissioning crypts in human tissue.
(TIF)

S4 Fig β4 Integrin signal intensity is not elevated in Paneth cells in *Apc* mutant organoids and tissue. (A) Polyps from *Apc*^*Min/+*^ mice and (C) *Apc*^*Min/Min*^ organoids were stained to visualise F-actin (Phalloidin, green), Lysozyme (white), and β4 Integrin (red). Lines in middle panels mark the boundaries between Paneth cells and their neighbours. Signal intensity of β4 Integrin is not elevated in Paneth cells in polyps (± SEM *p* = 0.0862, paired *t* test, *n* = 30) or in *Apc*^*Min/Min*^ organoids (± SEM *p* = 0.819, paired *t* test, *n* = 24) compared to neighbouring cells (S2 Data). (B′) In polyps, elevated β4 Integrin signal intensity is significantly reduced compared to in wild-type tissue (*p* = 0.0003, *t* test with Welch’s correction). (D′) In organoids, the ratio of β4 Integrin signal intensity is more variable and not significantly different from wild-type (*p* = 0.0638, *t* test with Welch’s correction). Underlying data for panels B, B′, D, and D′ can be found in S2 Data.
(TIF)

S5 Fig Muc2+ cells in the colon have higher β4 Integrin signal intensity on their basal surface than neighbouring cells. Projection of a colonic crypt sectioned and stained for F-actin (Phalloidin, green), Muc2 (white), and β4 Integrin (red) shows that Muc2-positive cells at the crypt base have more β4 Integrin on their basal surfaces than their neighbouring cells. Arrows in the top right panel point to CBCs neighbouring Muc2+ cells. White lines in the top left panel indicate the boundaries between Muc2+ cells and their neighbours.
(TIF)

S6 Fig Secretory cells in the small intestine have more β4 Integrin than neighbouring cells. Wild-type small intestinal crypts were sectioned and stained to visualise F-actin (Phalloidin, white), β4 Integrin (green), and secretory cells (red; Muc2 [A] and UEA-I [B]). White lines in the left panel indicate the boundary between secretory cells and their neighbouring cells. Both types of secretory cell have higher levels of β4 Integrin on their basal surfaces than neighbouring cells.
(TIF)

S7 Fig Physical constraint in tissue affects shape of fissioning crypts. Crypts stained to visualise nuclei (Hoechst, blue) and F-actin (phalloidin, red) show that daughter crypts in situ (A) elongate parallel to each other. When fissioning crypts are removed from tissue prior to fixing (B), daughter crypts adopt a more splayed conformation. In organoids (C), daughter crypts elongate at a 90°–180° angle. These observations suggest daughter crypts are kept parallel by physical constraint from surrounding tissue; when this physical constraint is released, daughter crypts adopt a more relaxed conformation. In organoids, daughter crypts elongate at larger angles due to the lack of constraint.
(TIF)

S8 Fig Ratio of β4 Integrin signal intensity on the basal surface of PC and CBC is similar in organoids and tissue. The levels of β4 Integrin on Paneth cells was measured relative to that on neighbouring CBCs (S2 Data). In both organoids and tissue, Paneth cells have approximately 1.3-fold more β4 Integrin on their basal surface than neighbouring CBCs (*p* = 0.7263, *t* test, *n* = 26 and 59 Paneth cells for organoids and tissue, respectively). Underlying data can be found in S2 Data.
(TIF)

S9 Fig Correlation between crypt length and number of Paneth cells. Correlation between crypt length and Paneth cell numbers (S4 Data) from organoids grown in ENR (A), ENR-CV (B), and ENR-CD (C). (D) Number of branches in organoids grown in ENR, ENR-CV, and ENR-CD at 24 h time intervals (S5 Data). Statistics shown were calculated by *t* test, and significance is compared to ENR control. Underlying data for panels A–C can be found in S4 Data, and for panel D in S5 Data.
(TIF)

S10 Fig Fissioning crypts have more Paneth cells and are longer. The length of crypts from organoids grown in ENR (A), ENR-CV (B), and ENR-CD (C) were measured and the number of Paneth cells (A′-C′) determined by counting (S4 Data). Fission was determined by crypt lumen bifurcation: if two crypt lumens meet, a crypt was scored as fissioning; a single crypt was defined as a crypt with a single base that did not open onto another crypt. Underlying data for all panels can be found in S4 Data.
(TIF)

S11 Fig Inhibitory Eph fragments do not alter the increased signal intensity of β4 Integrin on Paneth cells. The abundance of β4 Integrin on Paneth cells was measured relative to that on neighbouring CBCs (S2 Data). In both control organoids and organoids grown in the presence of inhibitory Eph fragments, Paneth cells have approximately 1.3-fold more β4 Integrin on their basal surface than neighbouring CBCs. These data indicate the Eph fragment does not affect cell-substrate adhesion (*p* = 0.2480, *t* test, *n* = 26 for ENR and 34 for Eph-treated). Underlying data can be found in S2 Data.
(TIF)

S12 Fig Proportion of Paneth cells affects the relative size of cells in the mathematical model. (A) Average cell area and (B) the relative cell area ratio (Paneth cell area ÷ Lgr5+ cell area) for varying Paneth cell numbers in the epithelial layer model (S10 Data). Here, Paneth cells are 4.5-fold stiffer than Lgr5+ cells (μ_p_/μ_s_ = 4.5). Error bars in (A) represent the standard deviation in cell area for Lgr5+ cells (blue) and Paneth cells (red). Underlying data for panels A and B can be found in S10 Data.
(TIF)

S13 Fig Mitosis occurs in the bifurcation of fissioning crypts. (A, B) Optical sections of a fissioning crypt stained against Hoechst (cyan) and Phalloidin (red). Mitotic cells were observed underneath the bifurcation, apparent by condensation of DNA (arrow). Imaris-rendered surfaces showing crypt lumen (red), crypt wall (transparent yellow), and PH3+ nuclei (green spots) from the bottom (C) and side (D) of a fissioning crypt. (E, F) Optical sections from tissue stained against PH3 (red) and Hoechst (cyan) reveal mitotic cells can be present both above and below the bifurcation in fissioning crypts.
(TIF)

S1 Table Percentage of fission during post-natal development. Percentage of crypts undergoing fission in all regions of the gut from mice aged 2, 3, 4, 5, 6, and 10 wk (see also S1 Data). Percentages of crypt fission are displayed in bold, standard deviations are displayed in italics. Underlying data can be found in S1 Data.
(TIF)

## Abbreviations

CBC: crypt base columnar
FAP: familial adenomatous polyposis
