# Supplementary figures and images for: Paneth Cell-Rich Regions Separated by a Cluster of Lgr5+ Cells Initiate Crypt Fission in the Intestinal Stem Cell Niche
Source: PLoS Biol. 2016 Jun 27;14(6):e1002491. doi: 10.1371/journal.pbio.1002491 (PMC4922642; doi:10.1371/journal.pbio.1002491)

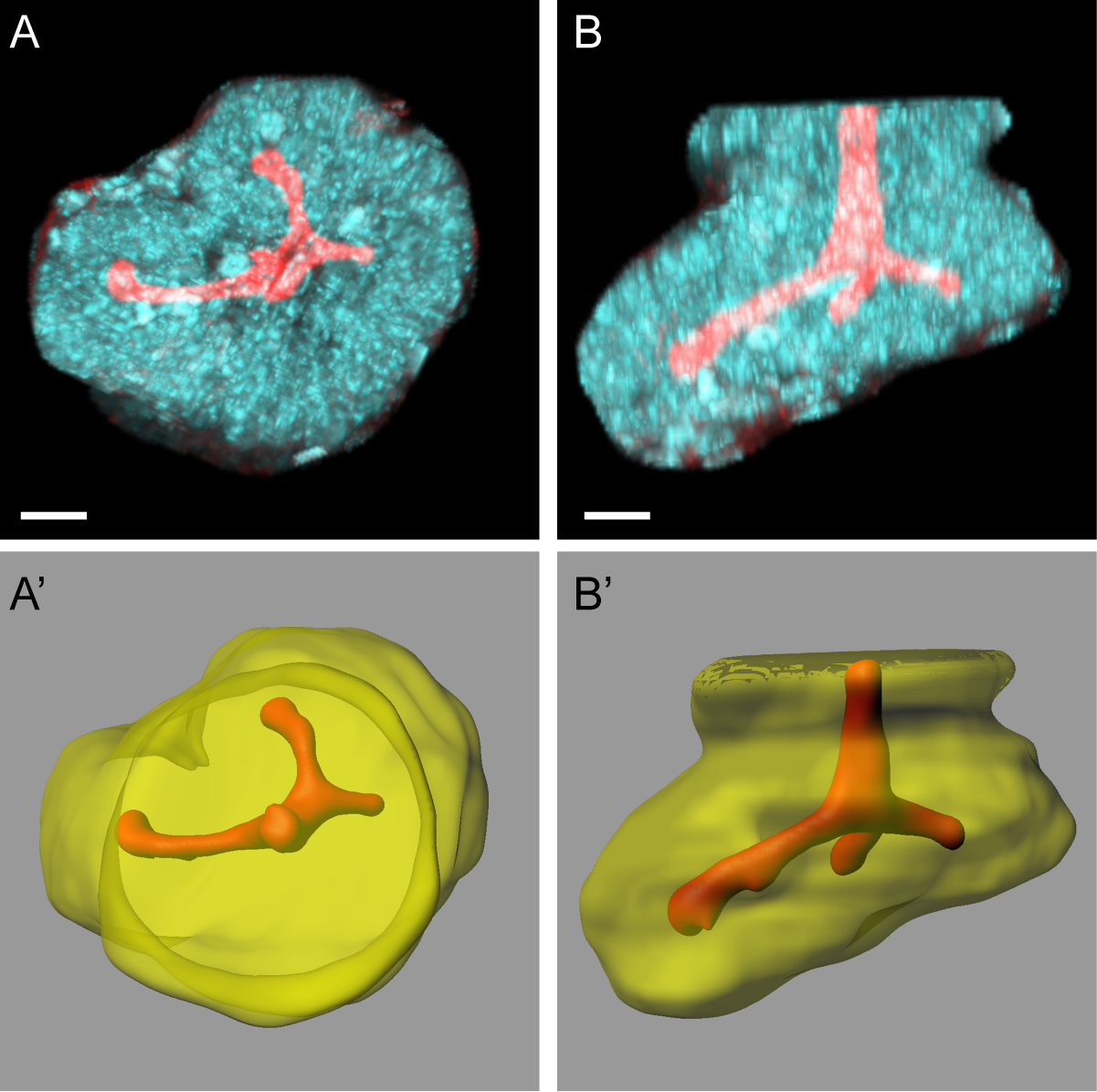

Supplement: S1 Fig — Crypts were prepared as in Fig 1 and show that in asymmetric triple fission, the crypt lumen trifurcates into three daughter crypts with at least one of different length. (A, B) 3D projections of Hoechst (cyan) and Phalloidin (red) stained tissue from the top (A) and side (B). (A′, B′) Imaris-rendered surfaces of the same crypt. In this example, one of the daughter crypts is shorter than the other two. (TIF) [file pbio.1002491.s013.tif]

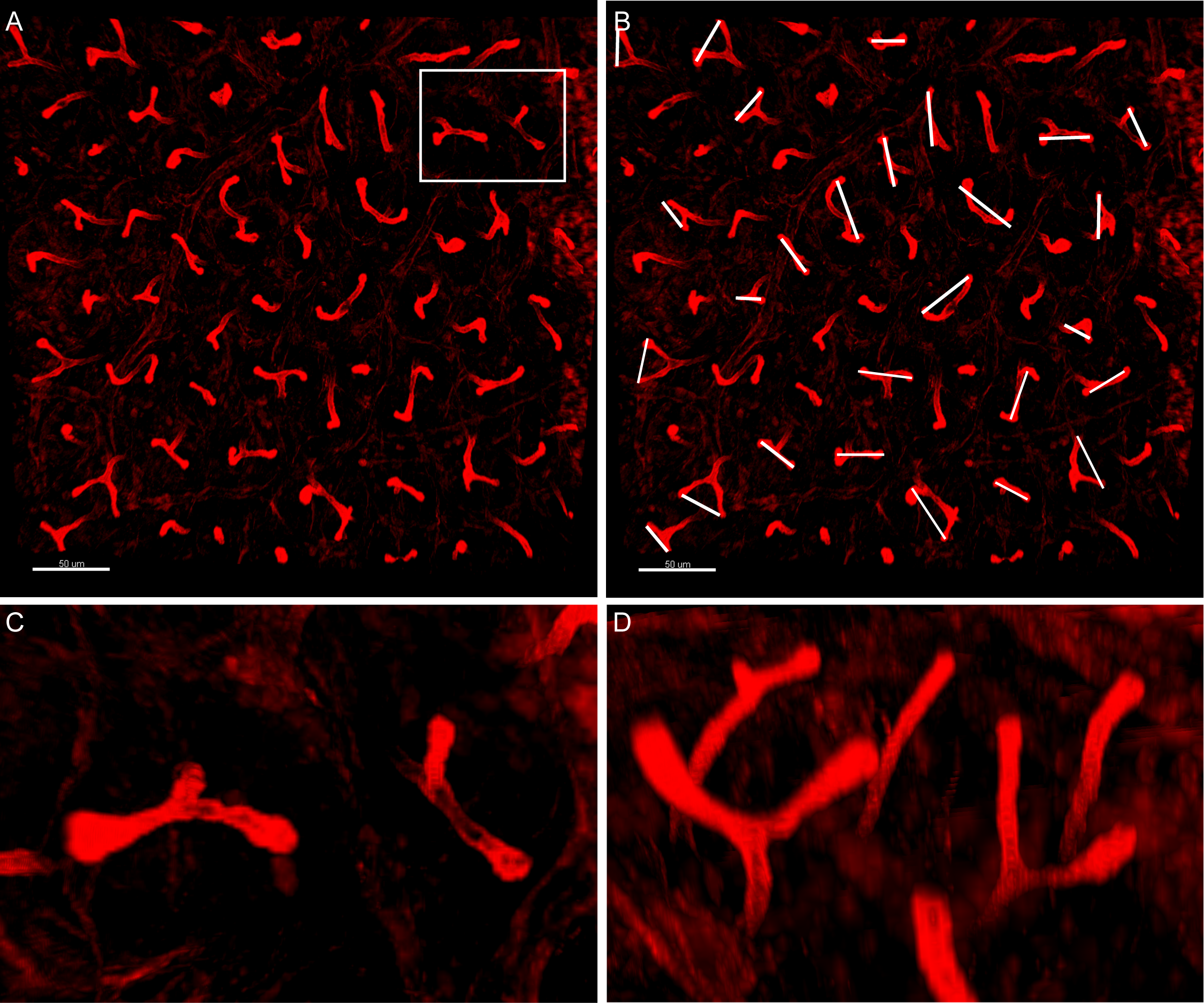

Supplement: S2 Fig — (A) Phalloidin stained small intestinal 3D whole mount tissue shows the lumens of crypts. (B) Highlighting crypt orientation by connecting the bases of daughter crypts shown in (A) reveals the diverse orientation of fissions. (C) Close-up and (D) side view of two adjacent crypts shows fissioning in different orientations (region highlighted by white box in [A]). (TIF) [file pbio.1002491.s014.tif]

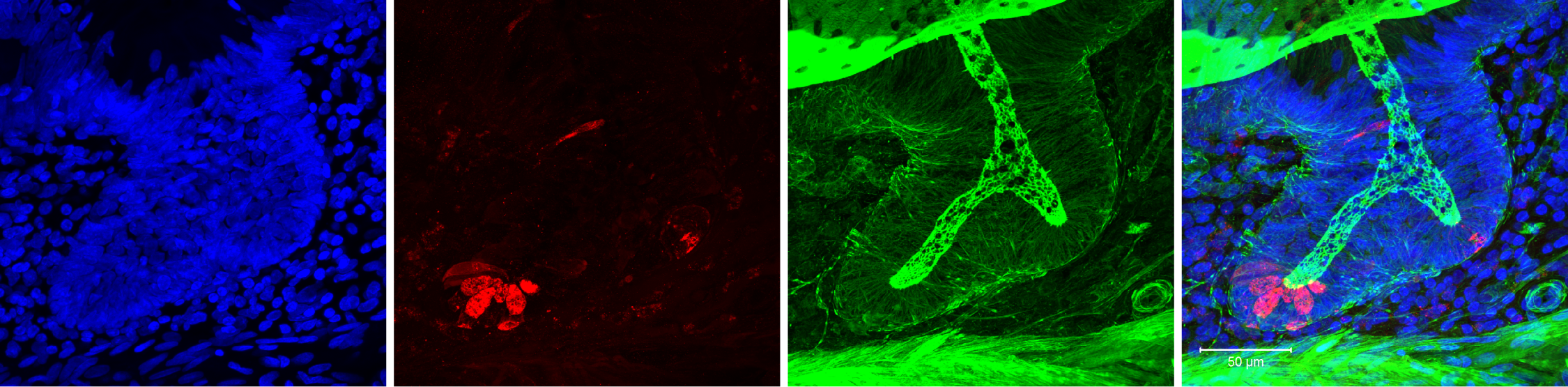

Supplement: S3 Fig — Fixed human tissue stained against Hoechst (blue), Lysozyme (red), and Phalloidin (green). Paneth cells are excluded from the region underneath the bifurcation in fissioning crypts in human tissue. (TIF) [file pbio.1002491.s015.tif]

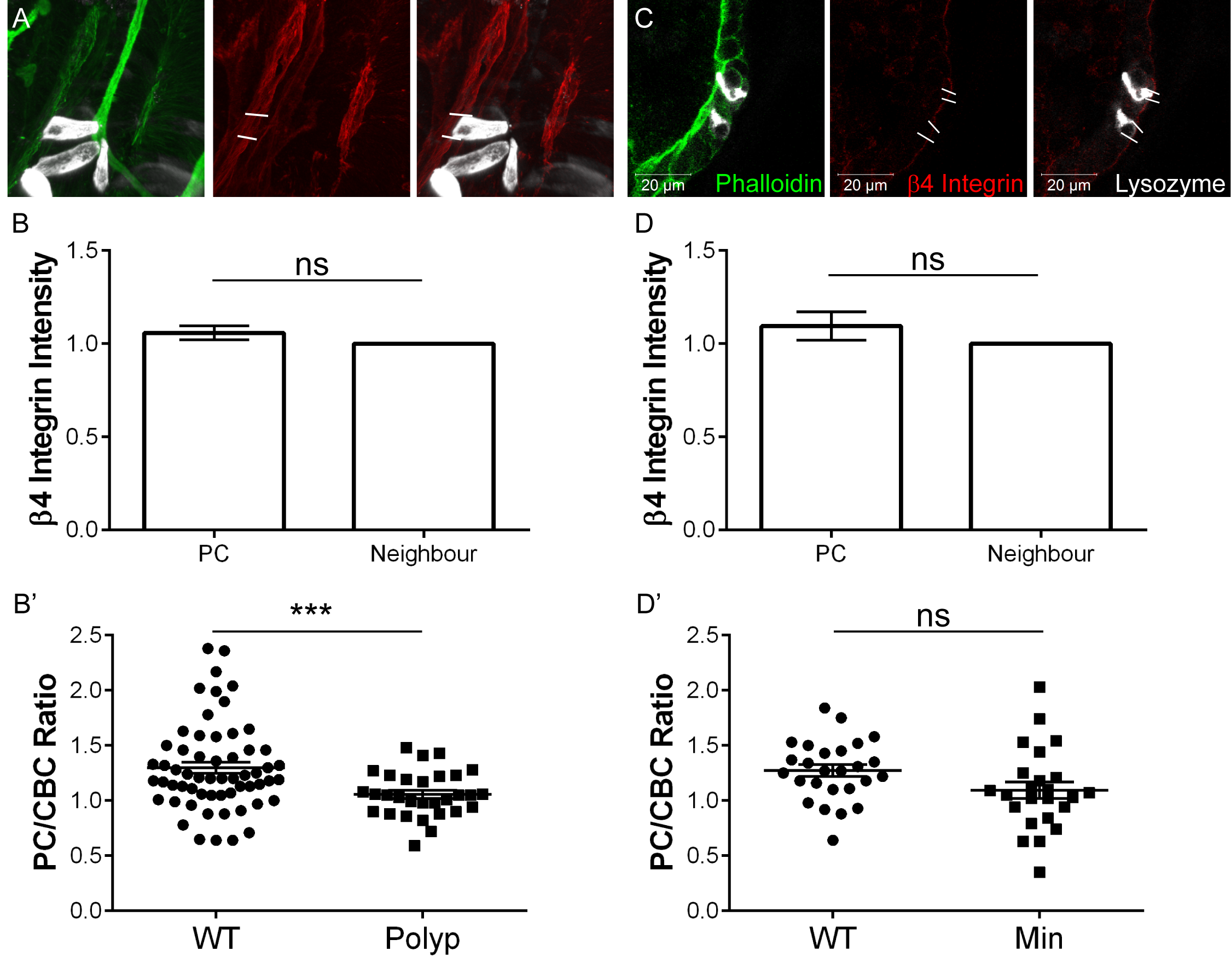

Supplement: S4 Fig — (A) Polyps from ApcMin/+ mice and (C) ApcMin/Min organoids were stained to visualise F-actin (Phalloidin, green), Lysozyme (white), and β4 Integrin (red). Lines in middle panels mark the boundaries between Paneth cells and their neighbours. Signal intensity of β4 Integrin is not elevated in Paneth cells in polyps (± SEM p = 0.0862, paired t test, n = 30) or in ApcMin/Min organoids (± SEM p = 0.819, paired t test, n = 24) compared to neighbouring cells (S2 Data). (B′) In polyps, elevated β4 Integrin signal intensity is significantly reduced compared to in wild-type tissue (p = 0.0003, t test with Welch’s correction). (D′) In organoids, the ratio of β4 Integrin signal intensity is more variable and not significantly different from wild-type (p = 0.0638, t test with Welch’s correction). Underlying data for panels B, B′, D, and D′ can be found in S2 Data. (TIF) [file pbio.1002491.s016.tif]

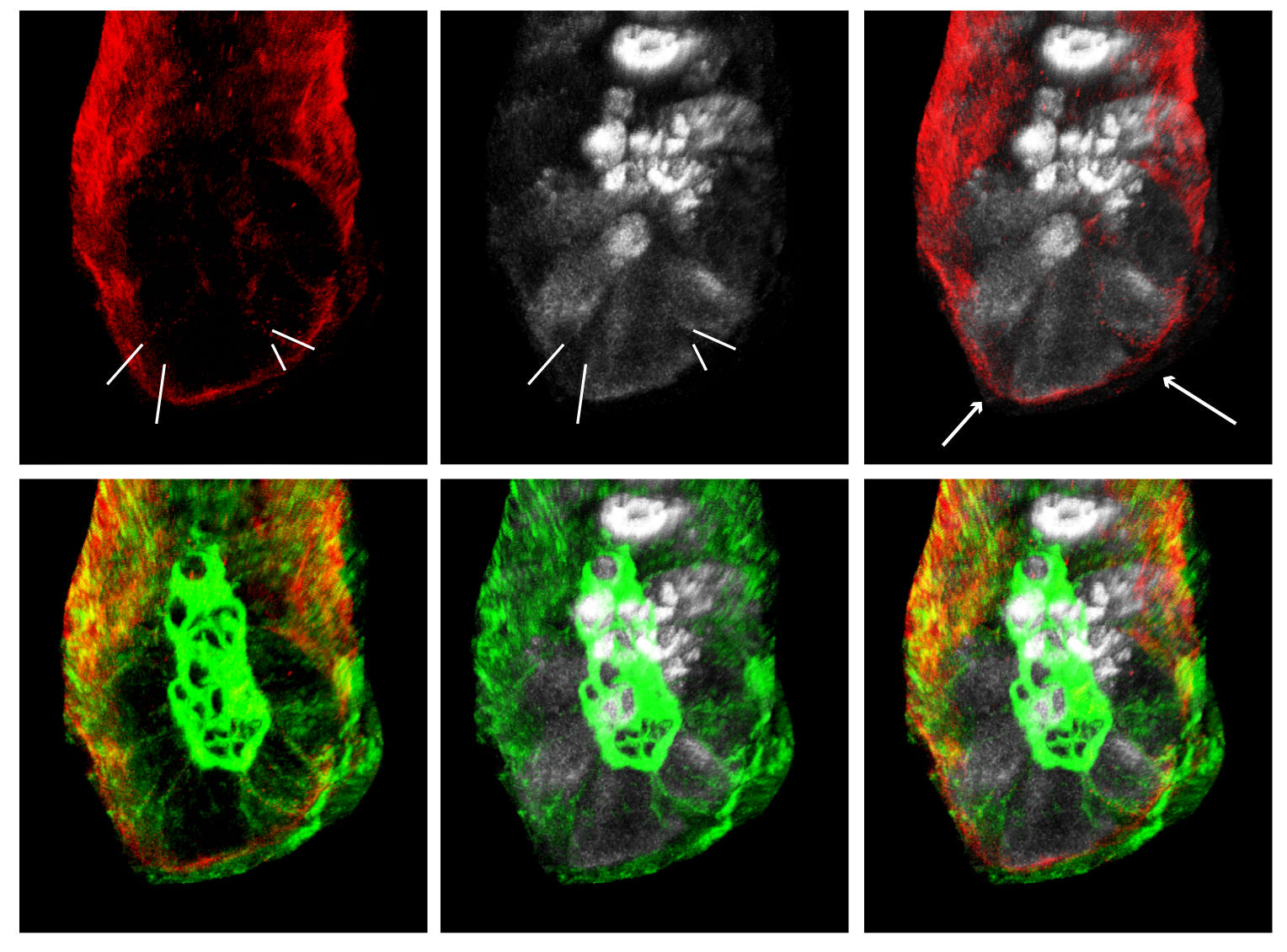

Supplement: S5 Fig — Projection of a colonic crypt sectioned and stained for F-actin (Phalloidin, green), Muc2 (white), and β4 Integrin (red) shows that Muc2-positive cells at the crypt base have more β4 Integrin on their basal surfaces than their neighbouring cells. Arrows in the top right panel point to CBCs neighbouring Muc2+ cells. White lines in the top left panel indicate the boundaries between Muc2+ cells and their neighbours. (TIF) [file pbio.1002491.s017.tif]

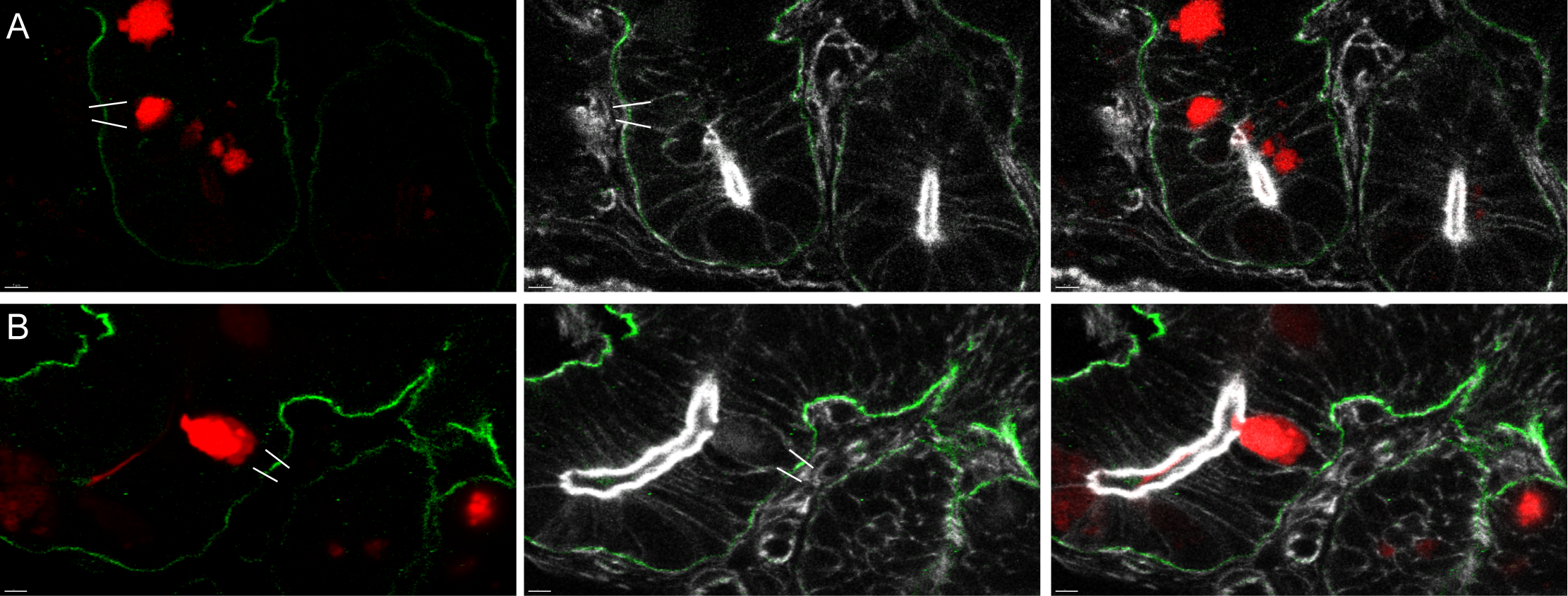

Supplement: S6 Fig — Wild-type small intestinal crypts were sectioned and stained to visualise F-actin (Phalloidin, white), β4 Integrin (green), and secretory cells (red; Muc2 [A] and UEA-I [B]). White lines in the left panel indicate the boundary between secretory cells and their neighbouring cells. Both types of secretory cell have higher levels of β4 Integrin on their basal surfaces than neighbouring cells. (TIF) [file pbio.1002491.s018.tif]

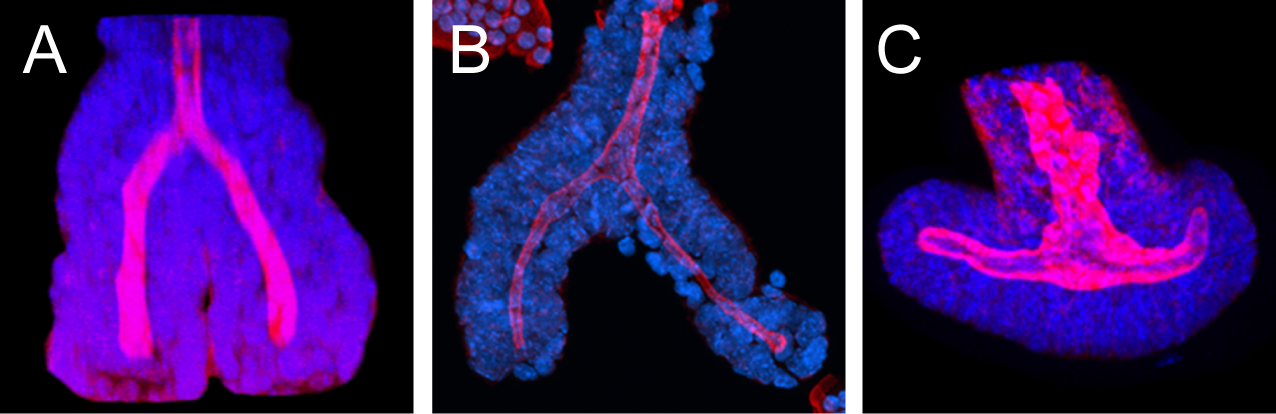

Supplement: S7 Fig — Crypts stained to visualise nuclei (Hoechst, blue) and F-actin (phalloidin, red) show that daughter crypts in situ (A) elongate parallel to each other. When fissioning crypts are removed from tissue prior to fixing (B), daughter crypts adopt a more splayed conformation. In organoids (C), daughter crypts elongate at a 90°–180° angle. These observations suggest daughter crypts are kept parallel by physical constraint from surrounding tissue; when this physical constraint is released, daughter crypts adopt a more relaxed conformation. In organoids, daughter crypts elongate at larger angles due to the lack of constraint. (TIF) [file pbio.1002491.s019.tif]

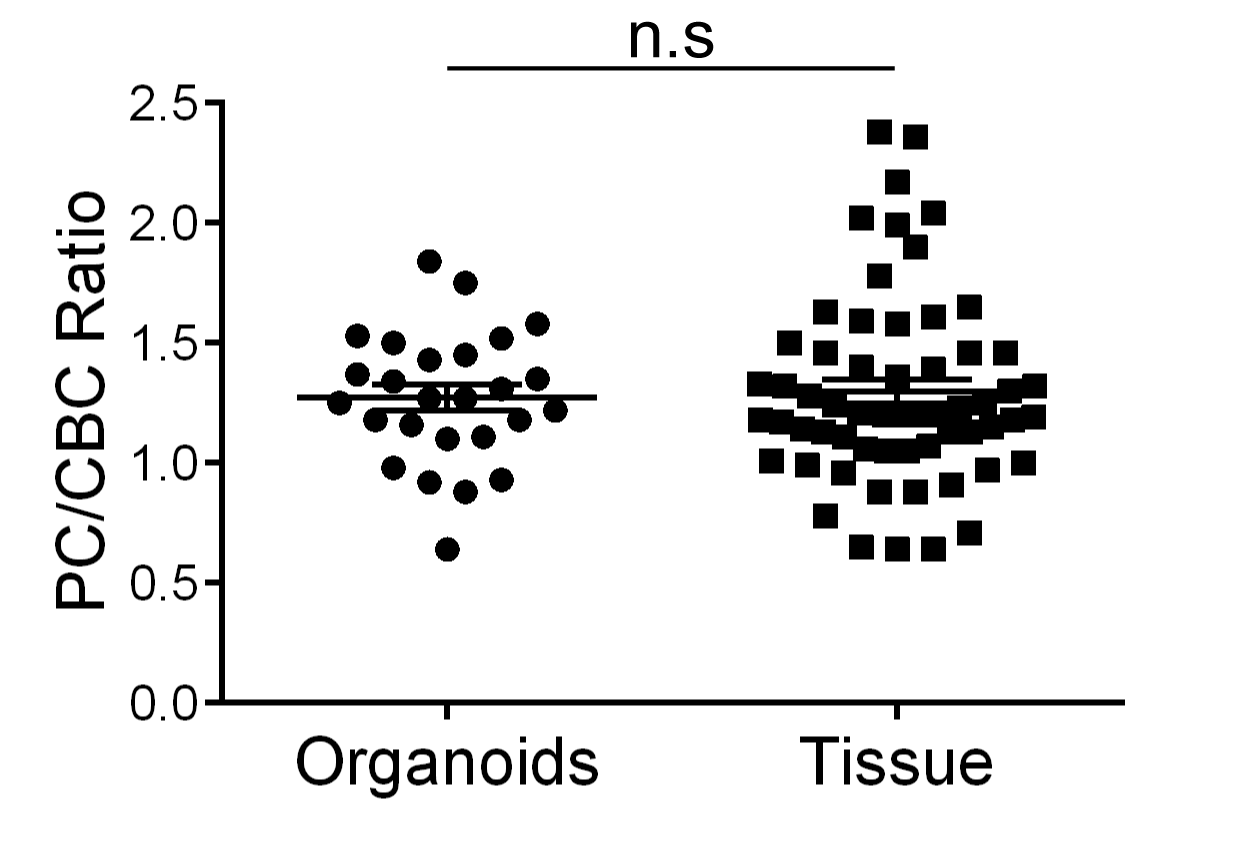

Supplement: S8 Fig — The levels of β4 Integrin on Paneth cells was measured relative to that on neighbouring CBCs (S2 Data). In both organoids and tissue, Paneth cells have approximately 1.3-fold more β4 Integrin on their basal surface than neighbouring CBCs (p = 0.7263, t test, n = 26 and 59 Paneth cells for organoids and tissue, respectively). Underlying data can be found in S2 Data. (TIF) [file pbio.1002491.s020.tif]

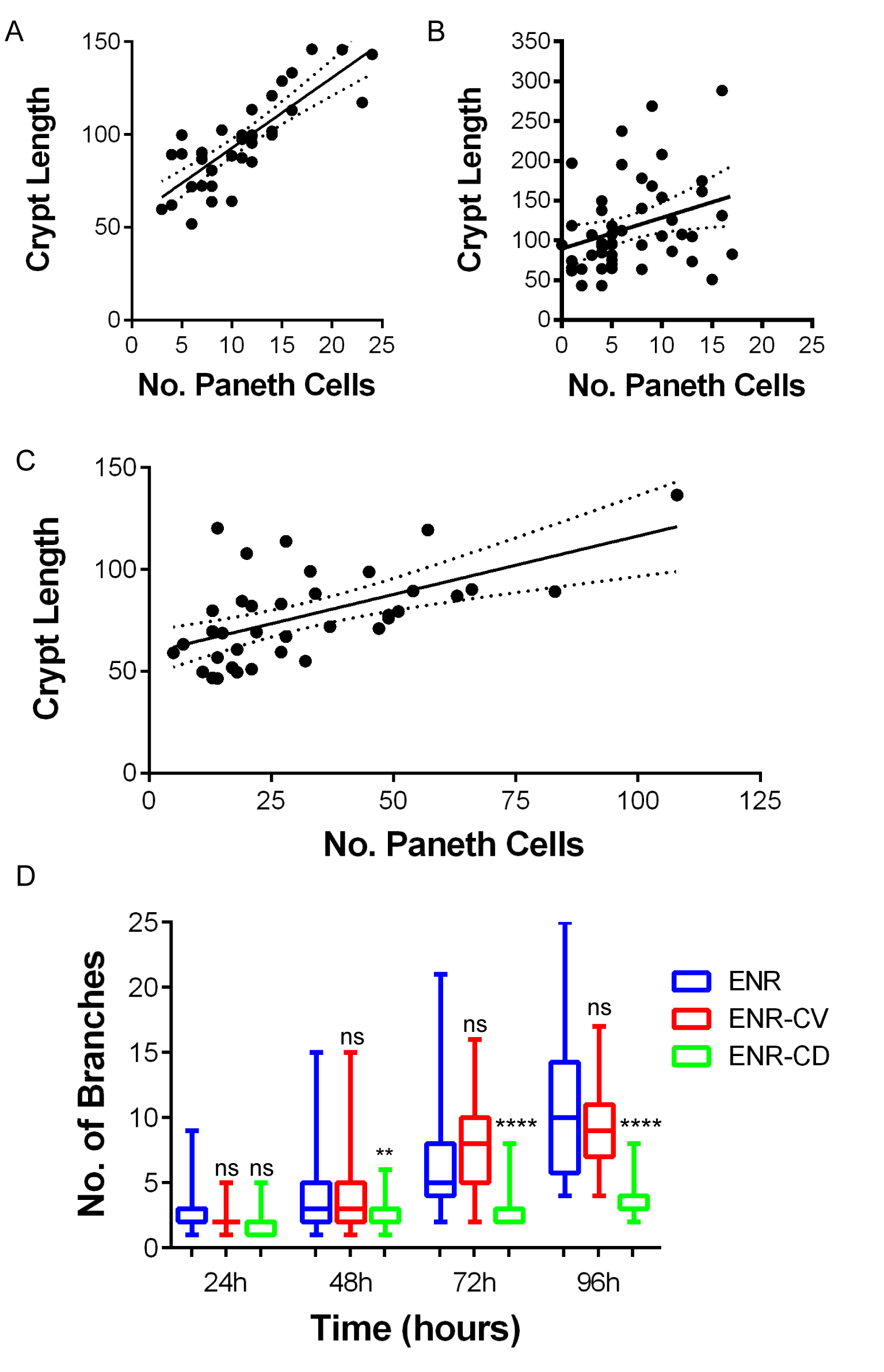

Supplement: S9 Fig — Correlation between crypt length and Paneth cell numbers (S4 Data) from organoids grown in ENR (A), ENR-CV (B), and ENR-CD (C). (D) Number of branches in organoids grown in ENR, ENR-CV, and ENR-CD at 24 h time intervals (S5 Data). Statistics shown were calculated by t test, and significance is compared to ENR control. Underlying data for panels A–C can be found in S4 Data, and for panel D in S5 Data. (TIF) [file pbio.1002491.s021.tif]

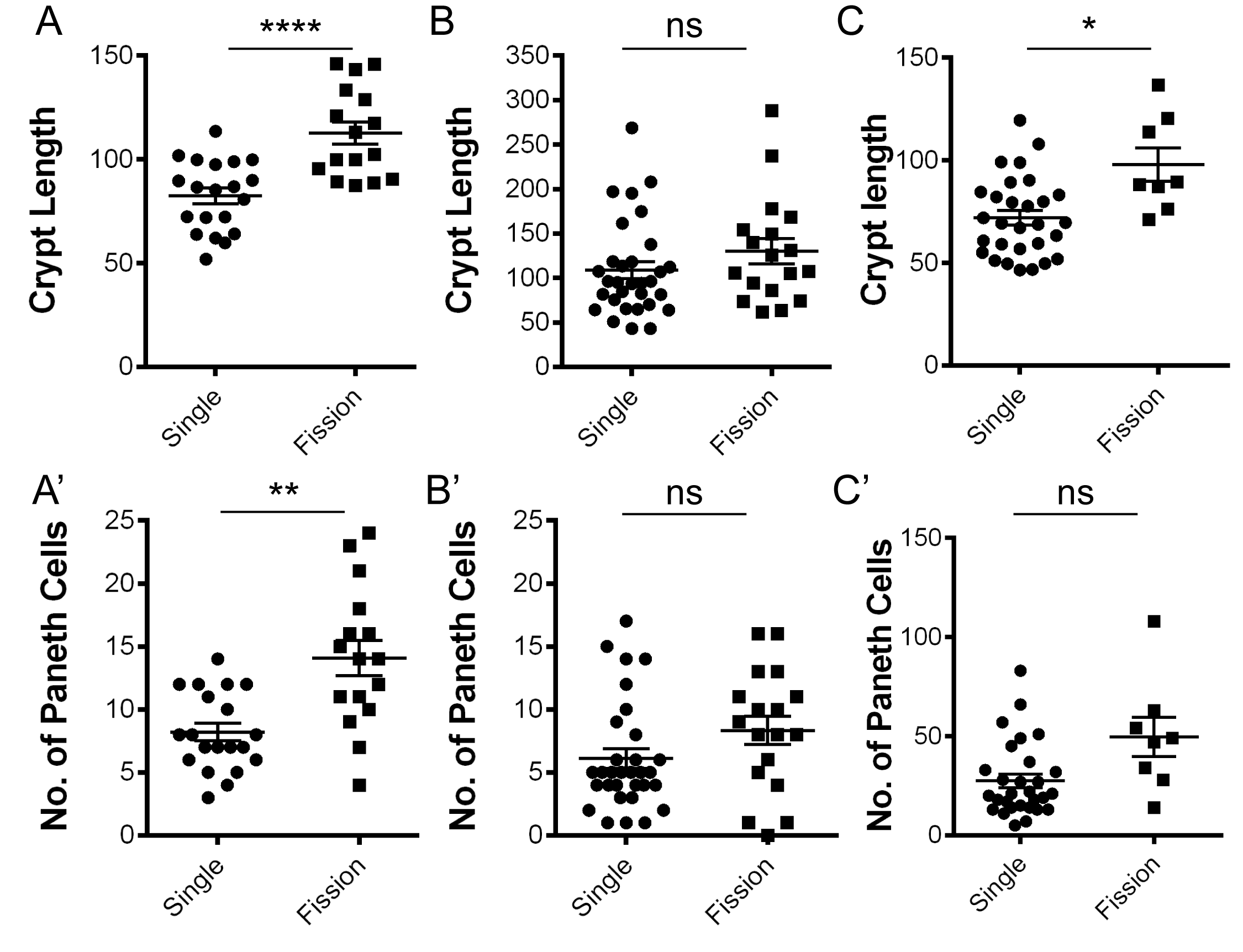

Supplement: S10 Fig — The length of crypts from organoids grown in ENR (A), ENR-CV (B), and ENR-CD (C) were measured and the number of Paneth cells (A′-C′) determined by counting (S4 Data). Fission was determined by crypt lumen bifurcation: if two crypt lumens meet, a crypt was scored as fissioning; a single crypt was defined as a crypt with a single base that did not open onto another crypt. Underlying data for all panels can be found in S4 Data. (TIF) [file pbio.1002491.s022.tif]

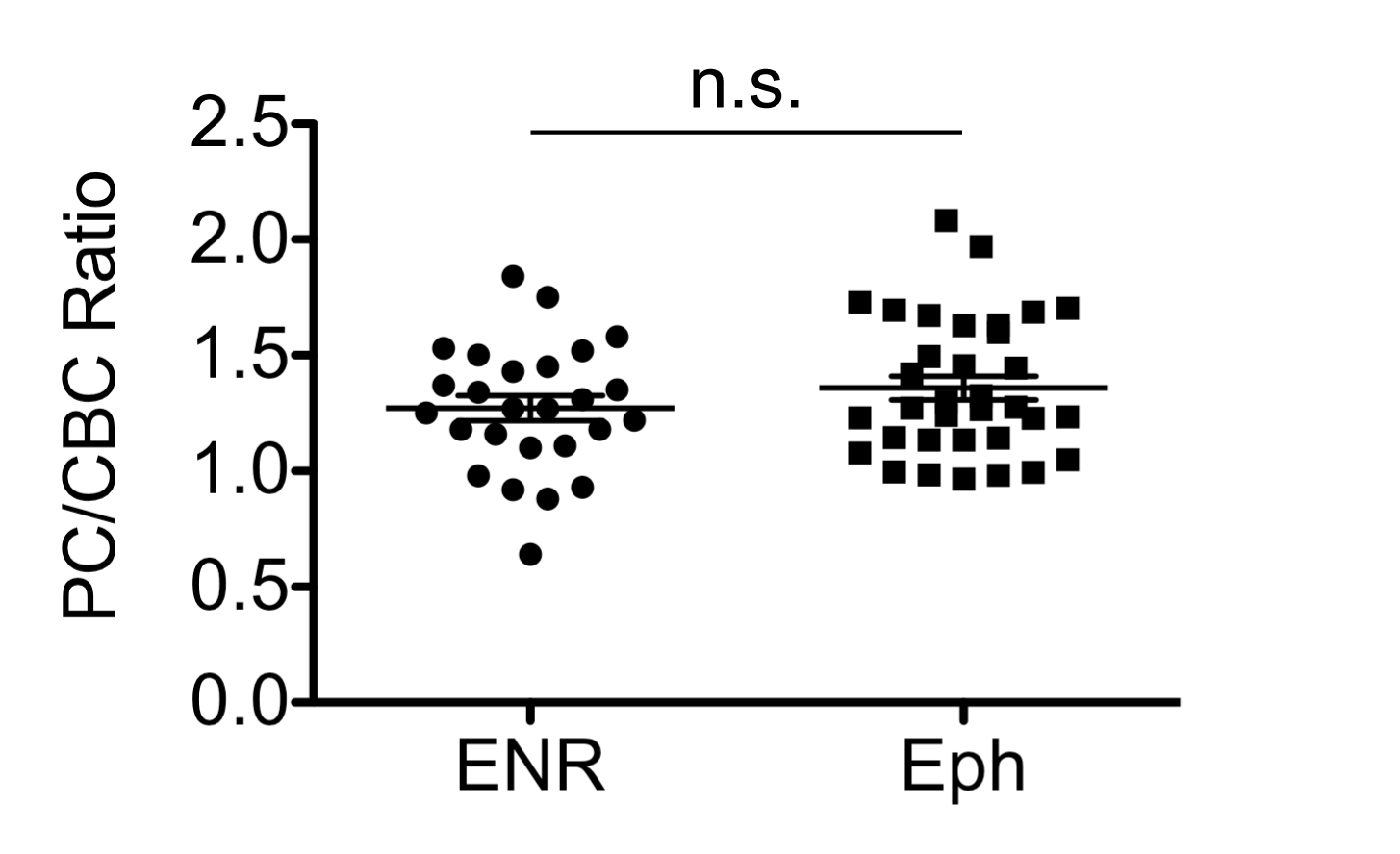

Supplement: S11 Fig — The abundance of β4 Integrin on Paneth cells was measured relative to that on neighbouring CBCs (S2 Data). In both control organoids and organoids grown in the presence of inhibitory Eph fragments, Paneth cells have approximately 1.3-fold more β4 Integrin on their basal surface than neighbouring CBCs. These data indicate the Eph fragment does not affect cell-substrate adhesion (p = 0.2480, t test, n = 26 for ENR and 34 for Eph-treated). Underlying data can be found in S2 Data. (TIF) [file pbio.1002491.s023.tif]

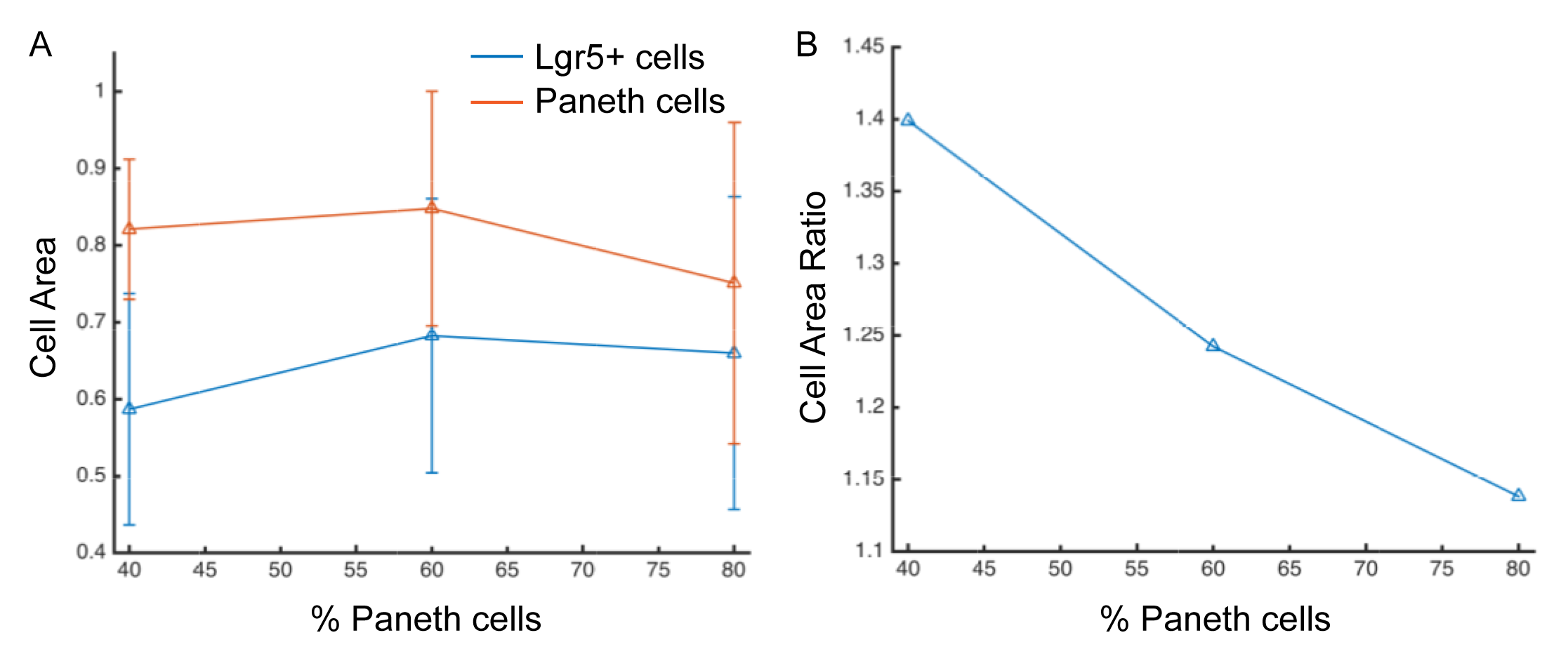

Supplement: S12 Fig — (A) Average cell area and (B) the relative cell area ratio (Paneth cell area ÷ Lgr5+ cell area) for varying Paneth cell numbers in the epithelial layer model (S10 Data). Here, Paneth cells are 4.5-fold stiffer than Lgr5+ cells (μp/μs = 4.5). Error bars in (A) represent the standard deviation in cell area for Lgr5+ cells (blue) and Paneth cells (red). Underlying data for panels A and B can be found in S10 Data. (TIF) [file pbio.1002491.s024.tif]

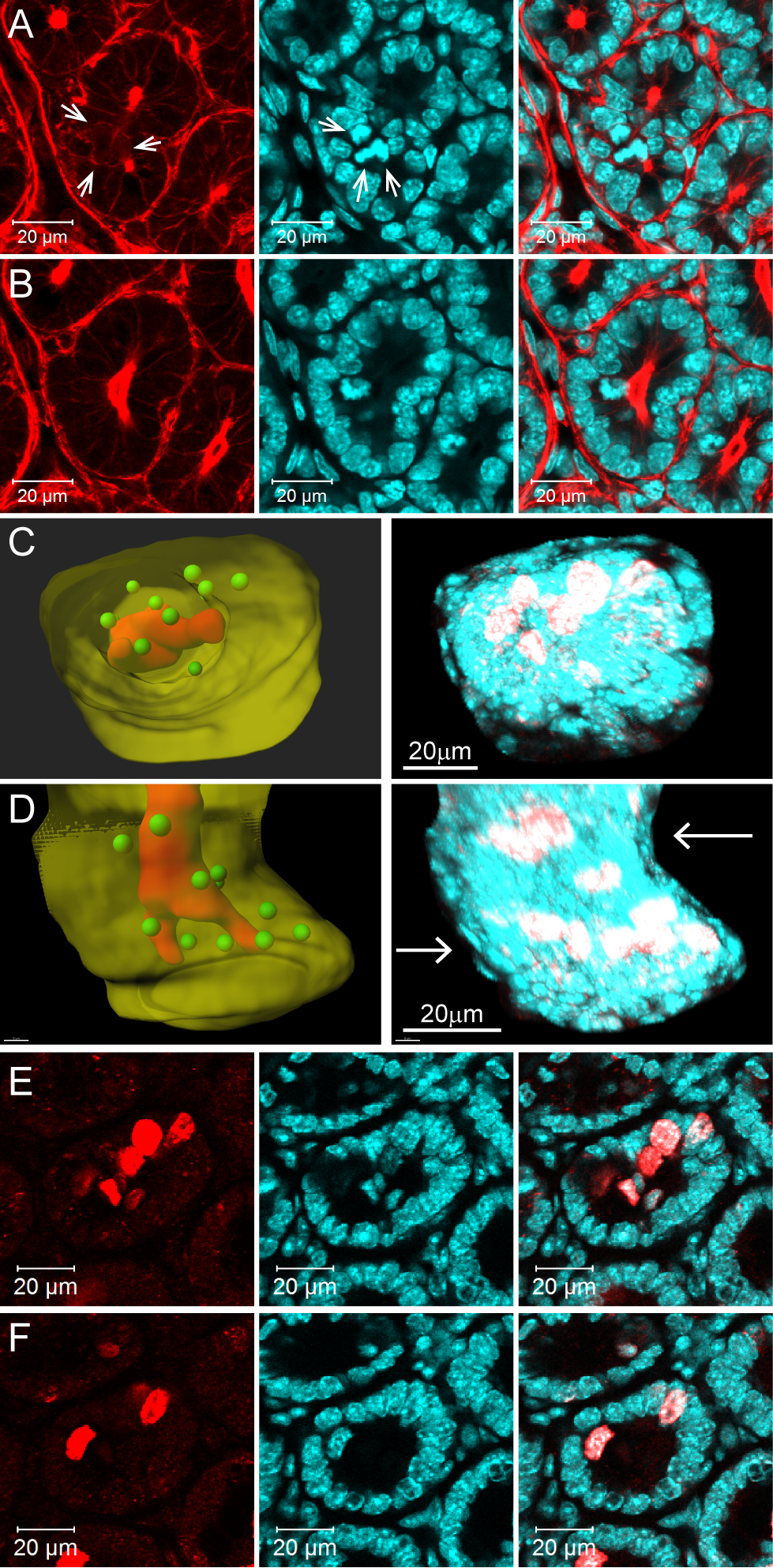

Supplement: S13 Fig — (A, B) Optical sections of a fissioning crypt stained against Hoechst (cyan) and Phalloidin (red). Mitotic cells were observed underneath the bifurcation, apparent by condensation of DNA (arrow). Imaris-rendered surfaces showing crypt lumen (red), crypt wall (transparent yellow), and PH3+ nuclei (green spots) from the bottom (C) and side (D) of a fissioning crypt. (E, F) Optical sections from tissue stained against PH3 (red) and Hoechst (cyan) reveal mitotic cells can be present both above and below the bifurcation in fissioning crypts. (TIF) [file pbio.1002491.s025.tif]

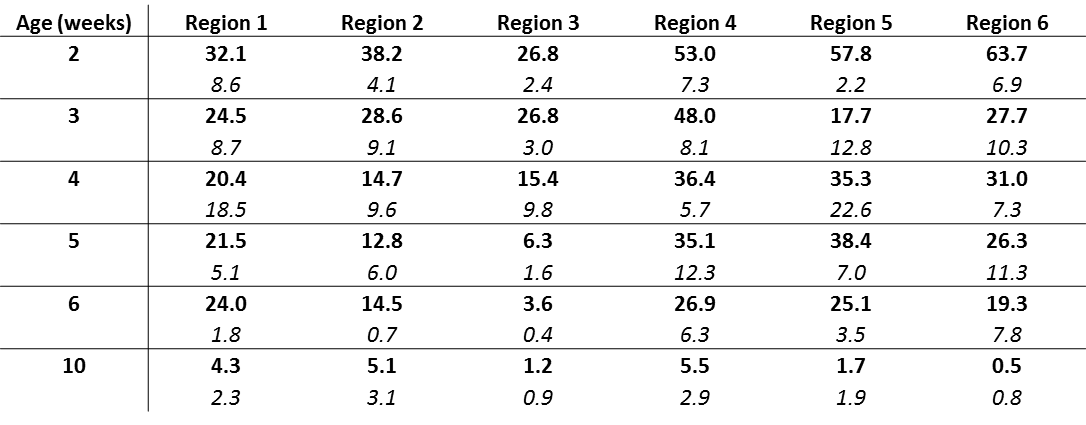

Supplement: S1 Table — Percentage of crypts undergoing fission in all regions of the gut from mice aged 2, 3, 4, 5, 6, and 10 wk (see also S1 Data). Percentages of crypt fission are displayed in bold, standard deviations are displayed in italics. Underlying data can be found in S1 Data. (TIF) [file pbio.1002491.s026.tif]
